# Dopamine‐Mediated Analog Control of Electrochromic Reactions Through Organic Electrochemical Transistor

**DOI:** 10.1002/smsc.202500635

**Published:** 2026-03-17

**Authors:** Giada D’Altri, Federica Mariani, Filippo Bonafè, Francesco Decataldo, Marta Tessarolo, Beatrice Fraboni, Erika Scavetta, Isacco Gualandi

**Affiliations:** ^1^ Department of Industrial Chemistry “Toso Montanari” University of Bologna Bologna Italy; ^2^ Department of Physics and Astronomy “Augusto Righi” University of Bologna Bologna Italy; ^3^ Department of Medical and Surgical Sciences University of Bologna Bologna Italy

**Keywords:** analog control circuit, dopamine electrochemical sensor, electrochromic actuation, organic bioelectronics, organic electrochemical transistor

## Abstract

The growing interest in bioinspired devices has led to a focus on bridging biological and electronic systems. Current bioelectronic devices predominantly rely on electrical signals and are unable to faithfully emulate the chemical signaling that plays a pivotal role in biological systems. To bridge this gap, we employ an organic electrochemical transistor (OECT) as the core of new platforms that integrates chemical sensing, analog computing, and electrochemical actuation in a single system. The OECT functions as a dopamine sensor. When a constant drain current is applied, dopamine oxidation at the gate electrode modulates both the drain voltage and the drain potential, as demonstrated by the measurements carried out with a reference electrode. These potential changes, usually exceeding 59 mV, are sufficient to significantly influence downstream electrochemical reactions. By fixing the drain current and gate voltage, the device enables tunable control over the electrochemical potential window. Actuation is demonstrated by electrically coupling the drain terminal to an electrochromic electrode, enabling direct and real‐time modulation of color in two different smart window materials: Prussian Blue and polyaniline. These results establish a versatile framework for chemical signal processing and actuation, enabling the next generation of adaptive bioelectronic interfaces that operate in chemical language.

## Graphical abstract

This article introduces an Organic ElectroChemical transistor (OECT) that enables analog electrochemical actuation directly coupled to dopamine sensing. Dopamine oxidation at the gate drives PEDOT reduction in the channel. Under constant drain current, the drain electrochemical potential shifts toward more negative values, triggering a continuous electrochromic response in a connected Prussian Blue electrode.

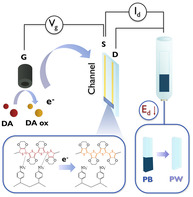

## 1 Introduction

Bioinspired devices have recently attracted significant attention from researchers, driven by the growing focus on human–machine interfaces (HMIs) and the pursuit of emulating biological systems to bridge the gap between the biological world and wearable, functional, and fast‐responsive technologies [1, 2, 3]. These bioelectronic systems have already demonstrated the ability to treat motor symptoms of Parkinson's disease [4] and to restore motor function following spinal cord injury [5], underscoring their transformative potential. However, despite these advances, current devices are largely limited to the transmission of electrical signals and are unable to replicate the chemical communication that is fundamental to the nervous system and mediated by neurotransmitters [6]. While recent progress in electrochemical sensing has enabled real‐time monitoring of biomarkers and environmental analytes [7, 8, 9, 10], the dynamic actuation of chemical responses in direct synchrony with detected signals remains a significant challenge. To address this unmet need, we investigated the potential of organic electrochemical transistors (OECTs) not only for highly sensitive detection but also for direct analog control of electrochromic reactions, thereby eliminating the need for intermediate digital data processing. Given the early stage of this technology, our proof‐of‐concept employs an electrochemical smart window [11] as the actuator, representing a significant step toward the seamless integration of chemical signaling within bioelectronic interfaces.

The choice of an OECT‐based sensing strategy arises from the unique versatility of this transistor architecture, which enables both sensitive detection and direct, dynamic modulation of channel conductivity to drive electrochemical processes [12]. OECTs feature the typical three‐terminal structure of a transistor‐source, drain, and gate, but differ from conventional field‐effect transistors in that the channel is separated from the gate by an ionically conductive electrolyte, instead of an insulating layer [13]. The gate electrode modulates the channel through an electrochemical reaction that typically involves the entire semiconductor volume, resulting in a high modulation, as demonstrated by typical transconductance values around 5 mS [14].

At the heart of the OECT lies an intrinsically conductive polymer channel, designed to undergo finely controlled redox reactions thanks to mixed ionic–electronic conduction [15, 16]. Materials such as polythiophenes [17], polypyrroles [18, 19], and polyanilines [20] have been widely explored, but PEDOT:PSS stands out for its remarkable stability and performance [21, 22]. Its molecular architecture, with negatively charged sulfonate groups stabilizing positive charge carriers and the ethylenedioxy bridge further delocalizing and protecting these charges, ensures robust operation. This intrinsic conductivity enables PEDOT:PSS‐based OECTs to operate in depletion mode [23], where the OECT elements can directly detect or be chemically modified so that the presence of biologically relevant analytes, such as glucose [24, 25], dopamine [26, 27], or glutamate [28, 29], can directly trigger electrochemical reactions that modulate device behavior in real time.

Moreover, OECTs are at the forefront of neuromorphic computing, being explored for the emulation of neural network features such as long‐term and short‐term plasticity [30, 31, 32]. Their integration into biocompatible, brain‐inspired devices is currently under investigation; however, their operating principle remains predominantly based on the use of electrical signals, while the exploitation of chemical signaling is still largely unexplored [33]. Remarkably, chemical signaling plays a pivotal role in the nervous system, as glutamate alone is responsible for mediating nearly 90% of all synaptic connections [34]. For instance, Santoro's group has pioneered the exploration of chemical neuromorphic modulation of artificial synapses based on OECTs [33, 35, 36]. Among their achievements is the development of an artificial synapse modulated by two neurotransmitters, whose behavior was investigated via optical modulation of PEDOT:PSS within the channel [35]. In a related approach, Bruno et al. introduced an organic platform integrated with silicon electronics for brain‐inspired computing, where adaptive synaptic potentiation and depression, which are induced by dopamine and H_2_O_2_, were leveraged to achieve real‐time control of a robotic arm [33]. OECT‐based circuits have also proven highly effective for advanced data processing. Wang et al. designed an OECT circuit capable of multimodal sensing, memory, and processing, enabling sophisticated functions such as conditional reflexes and even cardiac disease diagnosis [37]. Additionally, Fabiano's group engineered an electrochemical neuron composed of an OECT circuit, capable of processing frequency‐encoded signals and triggering movement in a Venus flytrap, thus bridging the gap between artificial computation and biological actuation [38]. In parallel, advances in scientific research have produced devices capable of precise chemical actuation, such as systems for controlled release of chemical species [39], on‐demand generation of reactive compounds [40, 41], and dynamic color modulation in electrochromic materials [42]. Collectively, these breakthroughs underscore the unique potential of OECTs to serve as a unified platform for sensing, computation, and actuation in bioinspired technologies.

The interest in combining sensing, computing and actuation is not limited to artificial synapses and neuromorphic circuits but also extends powerfully into pharmaceutical applications. On one hand, glucose‐responsive insulin pumps have already reached the market and hold great promise for improving diabetes management in millions of patients worldwide [43]. On the other hand, closed‐loop drug delivery systems [44], implantable devices for tailored chemotherapy [45], and smart antibiotic dispensers [46] represent innovative frontiers where real‐time sensor feedback dynamically regulates therapeutic interventions based on continuous biomarker monitoring. However, the sensing, processing, and actuating cores in these devices are different modules with mismatching features impacting on fabrication compatibility, integration density, data processing efficiencies, and integration in biological environments [37]. The ability to electrochemically detect a component in an electrolytic solution and simultaneously generate actuation within the same medium typically requires a complex circuit architecture that incorporates both electronic and ionic elements. A critical challenge in integrating electronic and electrochemical systems lies in their divergent reference frameworks: inducing electrochemical reactions requires applying potentials relative to a reference electrode (e.g., Ag/AgCl), while electronic circuits operate with ground‐referenced voltages. OECTs inherently diminish this mismatch, as their gate, channel, and source potentials can be also referenced to the electrolyte, enabling direct translation between ionic and electronic signals without external potentiostats. This unique property positions OECTs as versatile components for bioinspired systems that provide analog control.

In this work, we present a simple analog dopamine‐driven actuating system. While a recent work has demonstrated OECT‐based neuromorphic devices that interface with biological systems or emulate synaptic plasticity through neurotransmitter modulation [33], our approach uniquely focuses on direct analog control of electrochemical reactions, entirely bypassing complex digital processing. In our ionic circuit, the gate electrode is responsible for dopamine sensing, while the channel performs local data processing. An electrochromic actuator is directly connected to the drain electrode to exploit the potential drop occurring at channel that is visualized by color change. Dopamine oxidation at the gate is thus transduced into an electrochemical actuation using a single OECT, providing a proof‐of‐concept for seamless, analog integration of chemical sensing and actuation, with an enhanced ability of modulating the actuating potential. The ability to induce electrochromic changes in different materials demonstrates that the data processing and computing capabilities of the OECT structure can be effectively controlled by adjusting the applied sensing conditions.

## 2 Experimental Section

### 2.1 Materials

Clevios PH1000 suspension (PEDOT:PSS) was purchased from Heraeus. Ethylene glycol (EG), dodecyl benzenesulfonic acid (DBSA), and 3‐glycidoxypropyl trimethoxysilane (GOPS) were purchased from Merck. KH_2_PO_4_ 99.5–100.5%, LiClO_4_ 99.99%, KCl, 99.0–100.5%, and dopamine (DA) hydrochloride (named 3‐hydroxytyramine hydrochloride) ≥ 98% were all purchased from Sigma–Aldrich. FeCl_3_ · 6H_2_O 99% and K_3_Fe(CN)_6_ 99% were purchased from Riedel‐de Haën. The chemicals were used as received. Indium tin oxide (ITO) glass 150 × 150 × 1.1 mm was purchased from Delta Technologies. For its use, it was cut in 30 × 7 mm rectangular shapes. The glassy carbon electrode (GCE, *d* = 3 mm) was purchased from BASi, and the saturated calomel electrode (SCE) was bought from Amel.

### 2.2 Instruments

The potentiostat was Autolab PGSTAT204 potentiostat/galvanostat from Metrohm, operating with NOVA 2.10 program. Spectrophotometric measurements were conducted with 8453 Diode Array G1103A Spectrophotometer from HP/Agilent. B2902A Source meter from Keysight, with EasyExpert program, was used to characterize the transistor and to apply constant voltage and/or current.

### 2.3 Fabrication of PEDOT:PSS Channel

Glass substrates were cleaned by sonication in water and soap (10%)/acetone/isopropanol/distilled water baths. Then, 7 nm of chromium and 30 nm of gold were deposited by thermal evaporation, using a physical mask to define the OECT source and drain electrodes. Samples were subsequently covered with Kapton tape, and a laser cutter (Odin 22 from Thunder Laser) was used to selectively remove the tape regions corresponding to the OECT channels 6 × 3 mm obtaining a physical mask for PEDOT:PSS deposition. After an air plasma surface activation (15 W for 4 min), the PEDOT:PSS solution (94% PEDOT:PSS PH1000, 5% ethylene glycol (EG), 1% 3‐glycidoxypropyltrimethoxysilane (GOPS), and 0.25% 4‐dodecylbenzenesulfonicacid (DBSA)) was spin‐coated at 3000 rpm for 9 s. The resulting film thickness was (0.10 ± 0.01) μm. The samples were subsequently annealed at 120°C for 1 h, and Kapton tape was finally peeled‐off to define the OECT channel.

### 2.4 Preparation of Electrochromic Actuator Based on Prussian Blue

Prussian Blue (PB) was electrochemically deposited on ITO glass (area equal to 1 cm^2^) immersed in a solution containing 2.5 mM FeCl_3_, 2.5 mM K_3_Fe(CN)_6_ and 0.1 M KCl. The other electrodes in the three‐electrode cell were a SCE as a reference, a Pt wire as a counter electrode. The ITO glass was first polarized with an amperometry at +0.35 V for 20 s. PB was then electrochemically deposited through cyclic voltammetry (CV) between −0.2 and +0.4 V for 20 cycles at scan rate equal to 100 mV/s. After the first deposition, the ITO electrode was gently moved to remove the PB that did not perfectly adhere to the electrode surface. The deposition was repeated two other times to achieve a bright blue coloration [47].

The electrochromic behavior of PB ITO electrode was investigated with combined measurements using the CHI660C potentiostat and the spectrophotometer. A three‐electrode system was prepared inside a cuvette, in which the ITO glass with PB deposited was the working electrode, an Ag/AgCl the reference and a Pt wire the counter. Amperometry measurements were run at the potentials −0.4, −0.2, 0, +0.2, and + 0.4, while the spectra were registered after 60 s since the start of each measure.

Finally, CV was performed in 0.1 M PBS pH 7 and 0.1 M KCl to evaluate the capacity of the PB‐ITO electrode.

### 2.5 Characterization of OECT Behavior as Dopamine Sensor

The OECT is composed of a PEDOT:PSS channel, whose extremities were connected to the source and drain terminals of the source meter and the GCE connected to the gate terminal. The overall device was characterized by observing the transfer and output curves (Figure S1). In the transfer measurement, *I*_d_ varied by imposing *V*_g_ values between –0.5 and + 0.6 V with a 50 mV s^−1^ scan rate, while a constant + 0.3 V was applied at the channel. In outputs, *V*_g_ was varied from 0 to + 0.6 V, with an increment of + 0.1 V for each cycle, while *V*_d_ shifted from 0 to –0.5 V with a 50 mV s^−1^ scan rate. In the sensing system, the working electrode terminal of the potentiostat was attached to the drain connection of the source meter to provide a signal correlated to an electrochemical reference, as SCE. All the electrodes were immersed in 20.00 mL of phosphate buffer solution (PBS) 0.1 M, pH 7.00, with LiClO_4_ 0.1 M, under magnetic stirring. The experiments were carried out while: 1) the source meter applied a fixed gate voltage (*V*_g_) and a fixed drain current (I_d_), and measured the gate current (*I*_g_) and the drain voltage (*V*_d_) every 1 s; 2) the potentiostat acquired the open circuit potential of the drain terminal (*E*_d_) every 1 s. The source meter was set in floating mode. During the measurement concentrated DA solutions were added to the electrolyte to obtain a final DA concentrations of 1, 3, 10, 30, 100, 300, and 1000 µM in the cell. The values of *V*_d_ and *E*_d_ were employed to plot the calibration curves. The response time was the time necessary to reach 90% of the variation of the examined signals.

LOD values were calculated with the following equation:

(1) $$\mathrm{LOD}=\frac{3\times \sigma_{\text{blank}}}{m}$$

in which *σ*_blank_ represents the standard deviation of the blank, while *m* is the calibration curve's slope [48].

### 2.6 Setup for Electrochromic Control

The elements of the control system were the channel, the GCE, and the PB ITO electrode. The overall system is inserted in a cell containing 20.00 mL of PBS 0.1 M, pH 7.00, and KCl 0.1 M. The channel terminals were then attached to the source and drain contacts of Source meter, while the GCE was attached to the gate one. A second cable was connected from the drain extremity of the channel to the PB ITO electrode, forcing them at the same potential. To further avoid any secondary reaction between dopamine and PB, ITO electrode was inserted into a secondary cell separated by a septum from the main cell in which there was the OECT having 2 mL of the same solution. The imposed *V*_g_ and *I*_d_ values were optimized and chosen according to the results obtained, with final values of 0.5 V and –50 µA, respectively. Under magnetic stirring, a known addition of dopamine solution was made.

## 3 Results and Discussion

**FIGURE 1:**
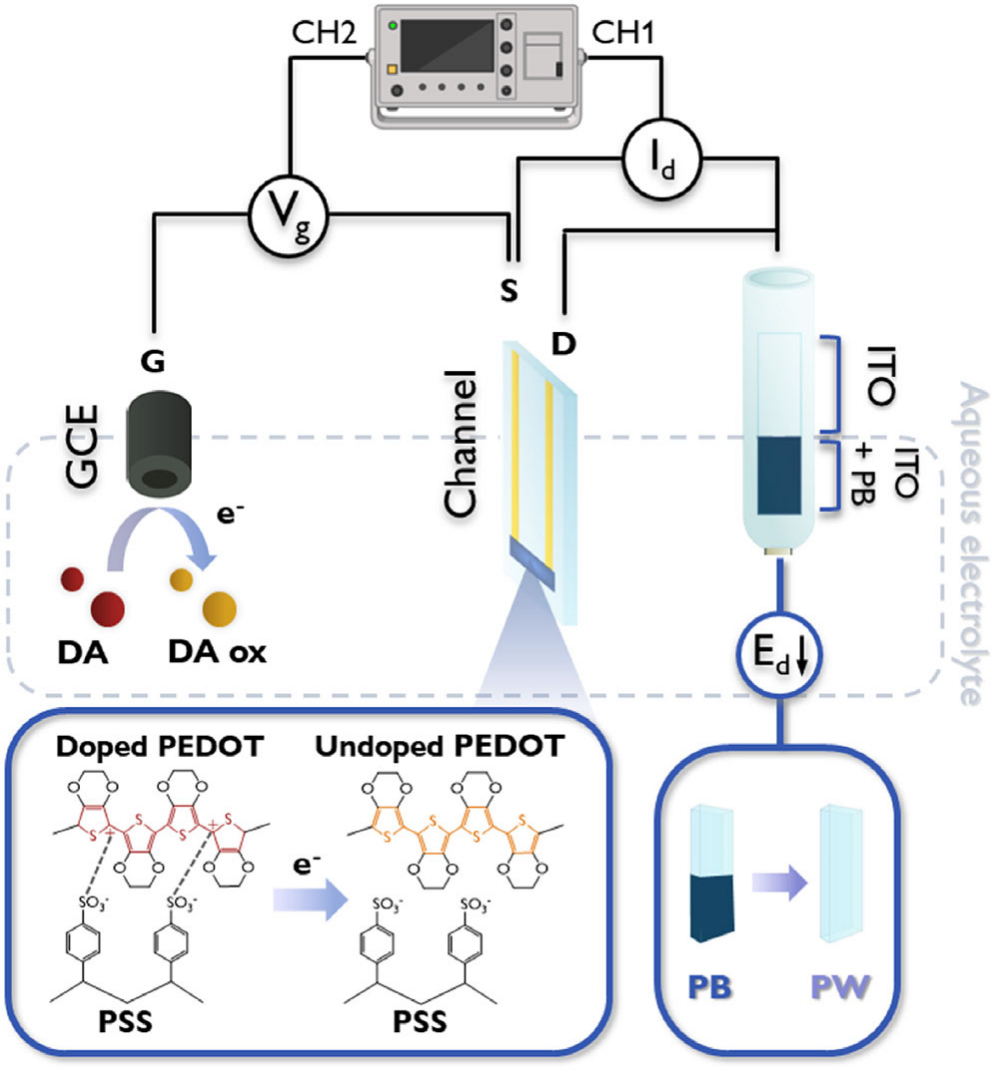
Schematics of the electrochemistry‐regulated phenomena enabling electrochromic control. The dopamine molecules are oxidized at the positively biased GCE and the resulting electrons dedoped PEDOT in the channel, where the consequent voltage variations actuate the electrochromic component (ITO) where Prussian Blue (PB) turns into Prussian White (PW).

Figure 1a illustrates the electronic–ionic hybrid circuit of dopamine‐driven actuation system, which employs an OECT as analog controller to modulate the electrochromic behavior of a PB‐coated ITO electrode, as a system integrated with sensing, computing, and actuating elements. The OECT is composed of a PEDOT:PSS channel of 6 × 3 mm^2^, whose capacitance was estimated by CV and results equal to 0.7 mF (Figure S2a) while its resistance is about 2.5 kΩ, and a GC (*d* = 3 mm) electrode as a gate. The PB‐ITO electrode, previously electrochemically characterized (Figure S2b), is electrically connected to the drain terminal of the OECT channel.

The gate electrode functions as a dopamine (DA) sensor through oxidative processes under positive gate voltage *V*_g_. Electrons liberated during DA oxidation are injected into the OECT channel, inducing a conductivity modulation that enables the precise triggering of electrochemical reactions in the chosen actuator. To operationalize this response, a constant channel current *I*_d_ is maintained via adaptive drain voltage *V*_d_ adjustment. However, electrochemical reactions are typically described as being governed by an electrochemical potential, here presented as the drain potential *E*_d_, which needs a relative reference to be expressed.

Our approach diverges from conventional fixed *V*_d_ methodologies by implementing constant *I*_d_ operation for DA detection while simultaneously monitoring *E*_d_ to quantify the electrochemical control ability. To confirm the opportunity of controlling an external device, an electrochromic actuator is then connected to the drain electrode terminal. These insights guided the final device design, enabling dual‐mode operation that integrates both sensing and actuation. This approach provides access to the range in which *E*_d_ varies, thereby allowing the device to modulate the occurrence of specific electrochemical reactions.

### 3.1 Dopamine Sensing through *V*_d_ Modulation

**FIGURE 2:**
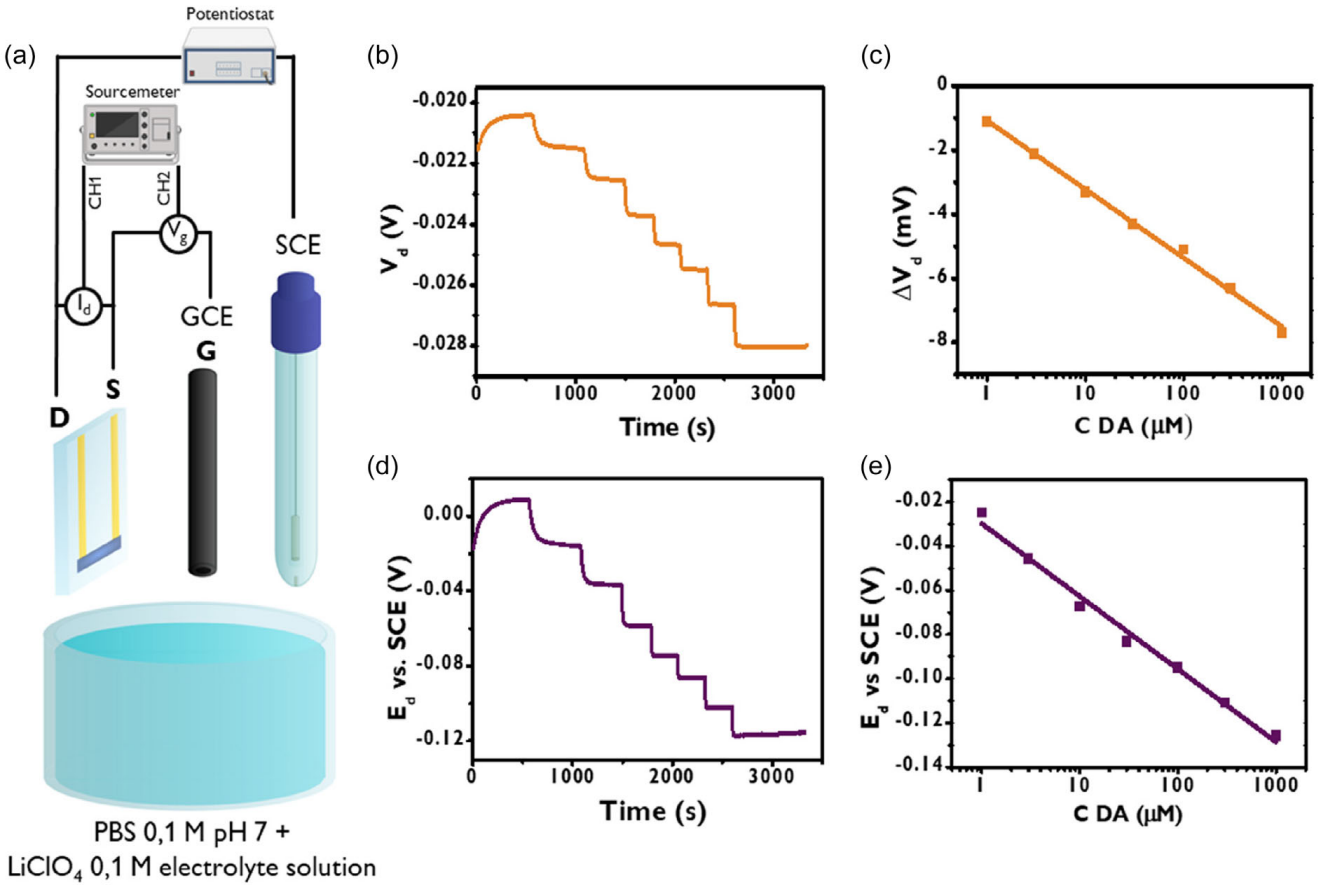
(a) Schematic structure of the electrochemical cell used for the acquisition of the calibration curves of dopamine. The electrochemical potential of the drain electrode (*E*_d_) is measured versus SCE through a potentiostat. (b) *V*_d_ versus time plot obtained with dopamine additions in the cell. (c) Corresponding Δ*V*_d_ versus C DA plot. (d) *E*_d_ versus time plot obtained during the acquisition of (b). (e) Corresponding Δ*E*_d_ versus C DA plot.

Previous studies have demonstrated dopamine detection using OECTs by monitoring *I*_d_ measurements [49]. In general, applying a positive *V*_g_ can induce dopamine oxidation at the gate electrode by reaching an electrochemical potential at the gate electrode *E*_g_ value sufficient to drive this reaction. The GC electrode was selected to ensure high reproducibility and electrochemical stability [50]. However, the dopamine‐induced modulation of *V*_d_ is yet to be optimized. With the aim of paving the path in this directions, the electrochemical potential of the drain terminal *E*_d_ was monitored as an open‐circuit potential using a potentiostat connected to a reference electrode that was immersed in the same solution, with the experimental configuration detailed in Figure 2a. Figure 2b exhibits *V*_d_ versus time trends obtained while different quantities of dopamine were added to the electrolyte (*V*_g_ = +0.200 V; *I*_d_ = −10 μA) to achieve controlled analyte concentrations over time. Each dopamine addition was detected by the OECT as an increase of absolute value of V_d_, which exhibits a negative value due to a negative imposed *I*_d_. Dopamine reacts with the positively biased glassy carbon gate electrode, following the reaction scheme [51]:

$$\text{Reaction 1 (gate)}:\mathrm{DA}\to {\mathrm{DA}}_{\mathrm{ox}}+2e^{‐}+2H^{+}$$

where DA and DA_ox_ are dopamine and oxidized form of dopamine, respectively.

This gate event induces PEDOT:PSS reduction in the channel, as schematically expressed by the well‐known reaction:

$$\text{Reaction 2 (channel)}:{\mathrm{PEDOT}}^{+}:{\mathrm{PSS}}^{‐}+M^{+}+e^{‐}\to {\mathrm{PEDOT}}^{0}+{\mathrm{PSS}}^{-}:M^{+}$$

The injection of cations (M^+^) from the electrolyte into the channel is balanced by the injection of electrons from the source/drain electrodes into the material bulk, thus maintaining charge neutrality.

The decrement of charge carrier concentration increases the resistance of the channel, and consequently a higher *V*_d_ is necessary to impose the fixed current. The signal time response exhibits a step‐like behavior, with *V*_d_ values reaching 90% of the final signal after 21 s. The OECT sensor configuration demonstrates a linear dependence of the drain voltage on the logarithmic dopamine concentration across three orders of magnitude (1 µM to 1 mM), as illustrated in Figure 2c. This relationship exhibits a slope of −0.00213 V decade^−1^ with a strong linear correlation (*R* ^2^ = 0.998), confirming V_d_ suitability as an analytical signal for dopamine quantification. Importantly, this logarithmic dependence aligns with the previously reported behavior for the drain current (*I*_d_) in OECT sensing [50], suggesting a consistent underlying mechanism for both voltage and current‐based detection approaches, which rely on a linear regime. Finally, the limit of detection (LOD) results equal to 0.1 μM.

**FIGURE 3:**
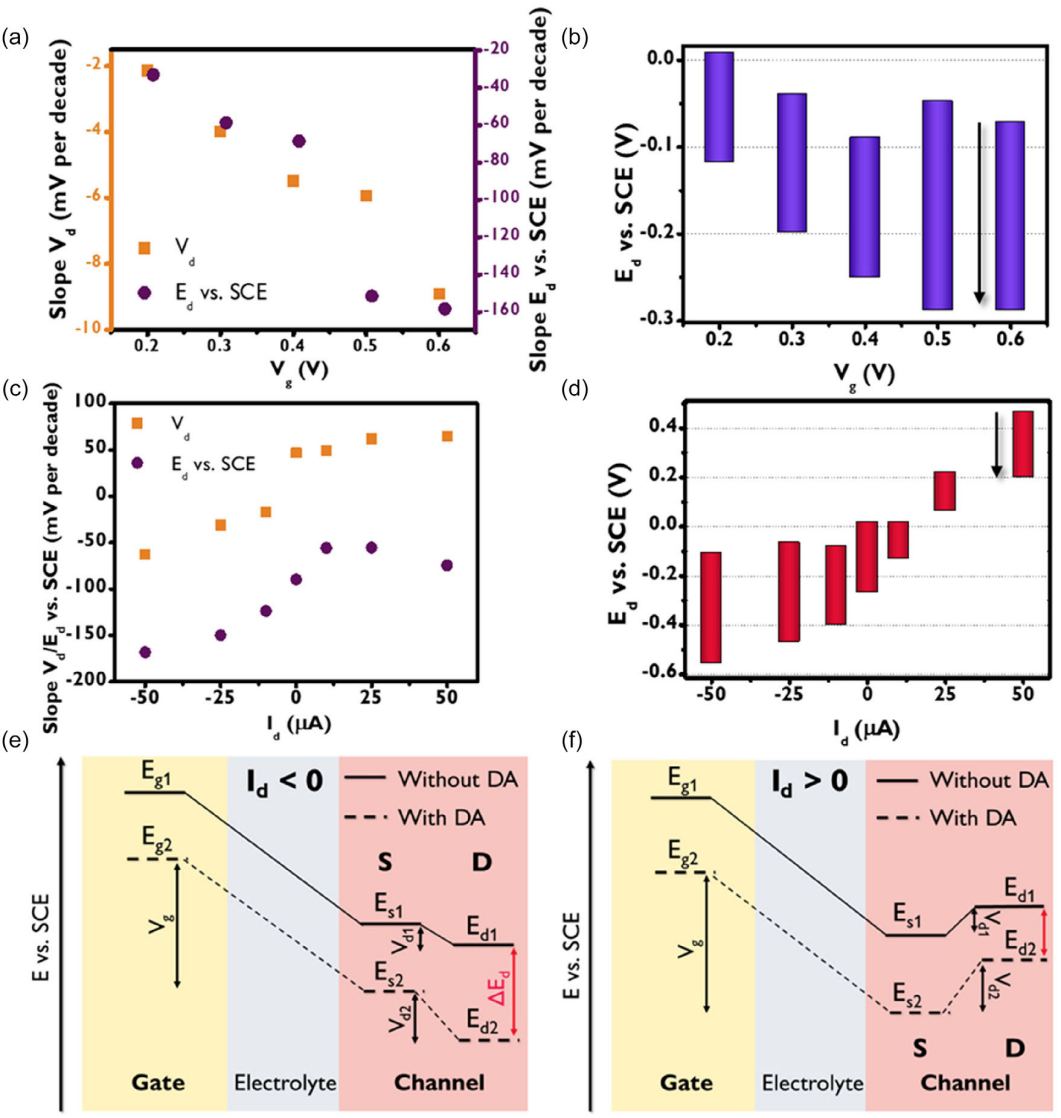
(a) Calibration curves’ slopes of *V*_d_ (orange) and *E* (purple) versus *V*_g_. (b) Correspondent *E* ranges in calibration curves by varying *V*_g_ from +0.2 to + 0.6 V with a constant *I*_d_ = −10 µA. The arrows indicate how the potential varies during the measurements. (c) Calibration curves’ slopes of *V*_d_ (orange) and *E* (purple) versus *I*_d_. (d) Correspondent *E* ranges in calibration curves by varying *I*_d_ from −50 to + 50 µA with a constant *V*_g_ = +0.5 V. (e) Scheme of energetic states variations in OECT system by applying a negative constant *I*_d_. (f) Correspondent scheme at positive constant *I*_d_.

Since the potentials were not applied versus a reference electrode, modulating *V*_d_ alone provides insufficient information for fine control of electrochemical reactions, because the source electrochemical potential, which serves as the reference for *V*_d_, varies during the experiment when measured against a true reference electrode. For a rigorous description of the thermodynamics governing the redox processes, the drain potential must be defined versus a reference electrode, as this potential directly controls the driving force for electrochemical reactions at the electrode–electrolyte interface. Under such conditions, the measured potential can be related to the electrochemical potential of electrons on an absolute scale, also referenced to electrons in vacuum, as discussed in the electrochemical literature, up to an additive constant [52]. For these reasons, we monitored *E*_d_ (the drain potential measured with respect to a saturated calomel electrode) throughout the measurement. Figure 2d shows the trends of *E*_d_ versus time during the experiment. As expected, each dopamine addition leads to a decrement of *E*_d_, which reaches a stable value after 50 s. The *E*_d_ variation is ruled by two different phenomena. First, all the voltages are related to each other, consequently when *V*_d_ decreases after dopamine additions, *E*_d_ must decrease accordingly. Moreover, the gate electrode extracts electrons from dopamine that are injected into the channel, dedoping it. Since electron injections occur throughout the entire channel from both source and drain electrode, thus reducing the concentrations of positive charges in the macromolecule, both *V*_d_ and *V*_s_ change their electrochemical potential (Figure 3e) and *V*_d_ is slightly affected as it is measured with respect to the source electrode. However, a stronger signal is detectable measuring *E*_d_ using an external reference electrode, such as a SCE, which is not influenced by these phenomena and has a specific value with respect to the vacuum level. The *E*_d_ values clearly exhibit a linear correlation to the logarithm of dopamine concentration, with slope and a *R* ^2^ of −0.033 V decade^−1^ and 0.988, respectively.

The LOD value resulted equal to 0.4 μM, when *E*_d_ is considered. Thus, sensors response is significantly higher when *E*_d_ is considered, since its potential value is anchored to the vacuum level. These outputs highlight that the system can synergistically work for driving electrochemical reactions.

### 3.2 Optimization of Dopamine‐Driven Actuators

During sensing, the OECT detects analytes through their oxidation, generating an ionic current that is actively processed and computed by the transistor architecture to yield dynamic *V*_d_ and *E*_d_ variations at the drain terminal. This computational capability transforms raw chemical information into precise, actionable signals, where the sensing conditions, such as applied potentials, imposed currents, and transistor geometry, all play a crucial role in shaping how the OECT interprets and responds to its environment. Below, we investigate the effects of *V*_g_ and *I*_d_ to showcase how the OECT's analog computing power can be harnessed to generate finely tuned *E*_d_ variations, enabling precise actuator control and opening new possibilities for intelligent, chemistry‐driven devices.

The sensing performance was first investigated by varying the applied *V*_g_ from + 0.200 to + 0.600 V, maintaining a fixed *I*_d_ of −10.0 μA (Figures S3–S6). Figure 3a shows the trends of slopes evaluated with *V*_d_ and *E*_d_ values vs logarithm of dopamine concentration in function of *V*_g_. Broadly speaking, the *V*_g_ increase leads to an increment of both *V*_d_ and *E*_d_ slopes, in agreement with a higher rate of dopamine oxidation induced by higher electrochemical potential values reached by the glassy carbon gate electrode. Figure 3a shows a magnification of the *E*_d_ slope when compared to *V*_d_ slope due to the low forced *I*_d_ current that does not generate high *V*_d_ values and enables uniform doping. Since the electrochemical actuation is ruled by the total *E*_d_ variation, Figure 3b shows the range of *E*_d_ values assumed when the electrolyte does not contain dopamine and for the same solution containing 1.00 mM of dopamine. The *E*_d_ values recorded at the experiment beginning exhibit a decreasing trend vs *V*_g_, due to the higher polarization generated by applied *V*_g_. At same time, the final *E*_d_ values show a decreasing trend until a plateau is reached for *V*_g_ values of + 0.500 and + 0.600 V. Interestingly, the highest *E*_d_ variation (−0.267 V) is observed for *V*_g_ value of + 0.500 V. The transfer curve exhibits a linear trend between *V*_g_ values of −0.5 and +0.6 V, indicating that the effect of the gate is essentially constant over the entire investigated potential range. In this framework, the OECT characteristics in the absence of DA cannot account for the enhanced performance observed at *V*_g_ = +0.5 V. This superior response is most likely due to the interaction between dopamine and the OECT‐based sensor. Therefore, the following experiments were carried out by applying a *V*_g_ equal to +0.500 V.

The influence of forced drain current *I*_d_ was systematically investigated by testing values ranging from −50 to +50 μA (−50, −25, −10, 0, +10, +25, +50 μA), as shown in Figures S7–S13. Figure 3c illustrates the relationship between the slopes of *V*_d_ and *E*_d_ versus logarithmic dopamine concentration across different *I*_d_ values, while the numeric values are shown in Tables S1–S3. Interestingly, the *V*_d_ slope maintained the same polarity as the applied current. The *V*_d_ variations show increasing values related to the values of negative forced *I*_d_, while the slope reaches a saturation at high positive *I*_d_ probably due to the high *V*_d_ values (also higher than *V*_g_) that induced dopamine oxidation also at the channel. By analyzing the *E*_d_ slope as a function of *I*_d_, it is evident that negative *I*_d_ values exhibit the highest *E*_d_ slope. This is because the negative variation in the electrochemical potential of the channel adds to the increase in negative *V*_d_ (Figure 3e), indicating that, under these conditions, all effects act synergistically to enhance OECT control. At the same time, the slopes of both *V*_d_ and *E*_d_ tend toward a minimum negative value as *I*_d_ becomes strongly negative. Such effect could be caused by the fact that the OECT enters the saturation operation regime, where the drain current is not controlled anymore by the drain voltage due to channel pinch‐off [53]. Conversely, when *V*_d_ is positive and *I*_d_ is also positive, the positive variation in *V*_d_ counteracts the negative variation in the channel's electrochemical potential (Figure 3f), thereby reducing the overall effect.

Figure 3d illustrates the variation of *E*_d_ observed during the sensing experiment. For negative *I*_d_ values, the highest *E*_d_ variation was recorded at an applied *I*_d_ of ‐50 μA, which is attributed to the significant contribution from *V*_d_ variation, as indicated by the steepest slope in Figure 3c. All data collected for negative *I*_d_ exhibit *E*_d_ variations that are relatively consistent with each other, ranging approximately from −0.10 to −0.50 V. However, imposing a positive *I*_d_ allows for greater control over the range and sign of *E*_d_ variation. For instance, when an *I*_d_ of +10 μA is applied, dopamine induces an *E*_d_ variation from 0 to −0.15 V, whereas for an *I*_d_ of + 50 μA, the *E*_d_ shifts from +0.45 to +0.20 V. To further validate the reproducibility of the system, measurements conducted with *I*_d_ = −25, −50 µA and *V*_g_ = +0.5 V were replicated thrice and are reported in Figure S14. The good reproducibility is highlighted by a percentage standard deviation of 6% and 9% for the DA concentration equal to 1 mM, respectively for –25 and −50 µA.

These findings indicate that varying positive *I*_d_ values can be exploited to change the range of *E*_d_ variation and achieve selectivity in the resulting E_d_. Crucially, each electrochemical process is governed by a distinct formal potential, which dictates the activation threshold and specificity of the reaction. Therefore, we must acknowledge the correlation between *E*_d_ variation and electrochemical actuation, in which the outcome cannot occur without the first element. Similarly, the design of the consequential dopamine‐driven actuation must consider that the discussed method operates with a decrease in *E*_d_ through PEDOT reduction in the channel. Thus, the dopamine‐driven actuation can induce *E*_d_ variations ranging between −0.148 and −0.447 V, corresponding to *I*_d_ values of +10 and −50 μA, respectively. From a thermodynamic perspective, an *E*_d_ variation of −59 mV could alter the equilibrium concentration ratio of reduced‐to‐oxidized species by a factor of 10 for single‐electron‐transfer processes. Kinetically, the same *E*_d_ variation could increase the rate of slow cathodic processes by tenfold. Consequently, dopamine‐induced *E*_d_ variations can effectively modulate and control electrochemical reactions in the actuation system. The validity of this approach will be demonstrated in next paragraphs by its application in regulating the color of an electrochromic material. An *I*_d_ of –50 μA was applied to maximize *E*_d_ variations, enabling precise control over the material's optical properties.

### 3.3 Preparation and Characterization of PB‐ITO Electrode

**FIGURE 4:**
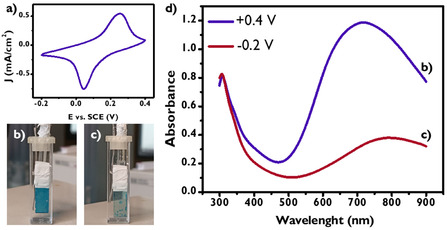
(a) Electrochemical deposition of PB through CV from −0.2 to +0.4 V versus SCE, with the resulting currents expressed over ITO glass area. (b) Three‐electrode cell in a cuvette after a first amperometry at +0.4 V versus Ag/AgCl for 60 s, with a blue PB‐ITO actuator. (c) Three‐electrode cell after a second amperometry at −0.2 V versus Ag/AgCl for 60 s, showing a blueish transparent actuator. (d) Absorption spectrum of PB deposited on ITO glass at +0.4 V (blue) and −0.2 V (red).

PB was selected as the electrochromic material due to its ability to undergo color changes within a potential range [54] that aligns with the *E*_d_ variation of the dopamine‐driven actuator. PB‐based materials played a key role in the development of smart windows [55], owing to their excellent electrochromic properties, chemical stability, and facile fabrication. PB was deposited on 7 × 30 mm ITO glass through CV within a potential window from −0.2 to +0.4 V, as shown in Figure 4a. To obtain a visible bright blue color, three different depositions were conducted on the same ITO electrode, 20 cycles each.

CV analysis of the PB‐modified ITO electrode (Figure S2b) exhibits a well‐defined reversible redox wave, corresponding to the electrochemical conversion between PB and PW through the reaction [56]:

$${\mathrm{MeFe}}^{\mathrm{III}}\left[{\mathrm{Fe}}^{\mathrm{II}}{\left(\mathrm{CN}\right)}_{6}\right]+{\mathrm{Me}}^{+}+e^{‐}⇄{\mathrm{Me}}_{2}{\mathrm{Fe}}^{\mathrm{II}}\left[{\mathrm{Fe}}^{\mathrm{II}}{\left(\mathrm{CN}\right)}_{6}\right]$$

#### 3.3.1 Blue Uncolored

Its electrochromic behavior has been confirmed through spectroelectrochemical investigation. The measurements were carried by subjecting the actuator to amperometry at different potentials, inducing color change, and triggering the spectrophotometric analysis after biasing for 60 s. The color change evaluation emerges from the comparison of Figure 4b,c, with the first presenting PB in its oxidized state with blue coloration when ITO glass was polarized at +0.4 V versus Ag/AgCl (Figure 4b), while the second (Figure 4c) demonstrates the electrochromic behavior induced by PB reduction to PW at −0.2 V vs. Ag/AgCl, characterized by an almost transparent and colorless state. Furthermore, the variation is quantitatively reported in spectrophotometric analysis shown in Figure 4d, in which the PB spectrum (+0.4 V) shows an absorption peak at 700 nm, while the PW spectrum (−0.2 V) has a visible decreased absorption at the same wavelength.

However, to achieve the desired control, the electrochemical process occurring in OECT sensing system must force electrochemical reactions in the actuator. Therefore, the dopamine‐driven actuator was designed to have a capacitance of the actuator close to the channel capacity. Both capacitances were estimated by CV (see Supporting Information). Since the channel capacity was 0.7 mF, while the specific capacitance of PB ITO electrode was measured as 12 mF cm^−2^, the PB area used in dopamine‐driven actuator was reduced to 0.1 cm^2^ to have a PB‐ITO electrode capacitance of 1.2 mF.

### 3.4 Dopamine‐Driven System Assembly

As the various elements of the designed system were characterized, the final dopamine‐driven actuation system can be assembled. The preparation of the cell requires both the channel and as the GC gate electrode, resembling the OECT needed for the sensing and computing phases. PB is then employed as a smart window actuator, thus responding to *E*_d_ variation in input. Consequently, the PB‐ITO electrode responds to *E*_d_ decrease, imposed by PEDOT reduction, with a color change provided by PB reduction to PW. Since the overall system relies on dopamine oxidation, parasite secondary reactions could occur between PB and dopamine itself. To avoid this and purposely showcase the capability of the proposed mechanism of inducing the desired actuation, the PB‐ITO electrode was separated from the system by a septum, while also being immersed in PBS 0.1 M pH 7.00. The septum enables electrochemical reactions to occur by allowing charge transfer across it while maintaining distinct compositions in the sensing system and the smart window compartments. In particular, a blank experiment confirming the need for a septum was conducted by adding dopamine to the PB‐ITO electrode immersed in PBS 0.1 M pH 7.00 under magnetic stirring, as showed in Multimedia File 1 (SI), while in Multimedia File 2 (SI) was shown how the presence of the septum avoids the color change. This experiment highlighted the need to separate the chromic actuator, while also proving that throughout *E*_d_ variation an enhanced color change is provided.

**FIGURE 5:**
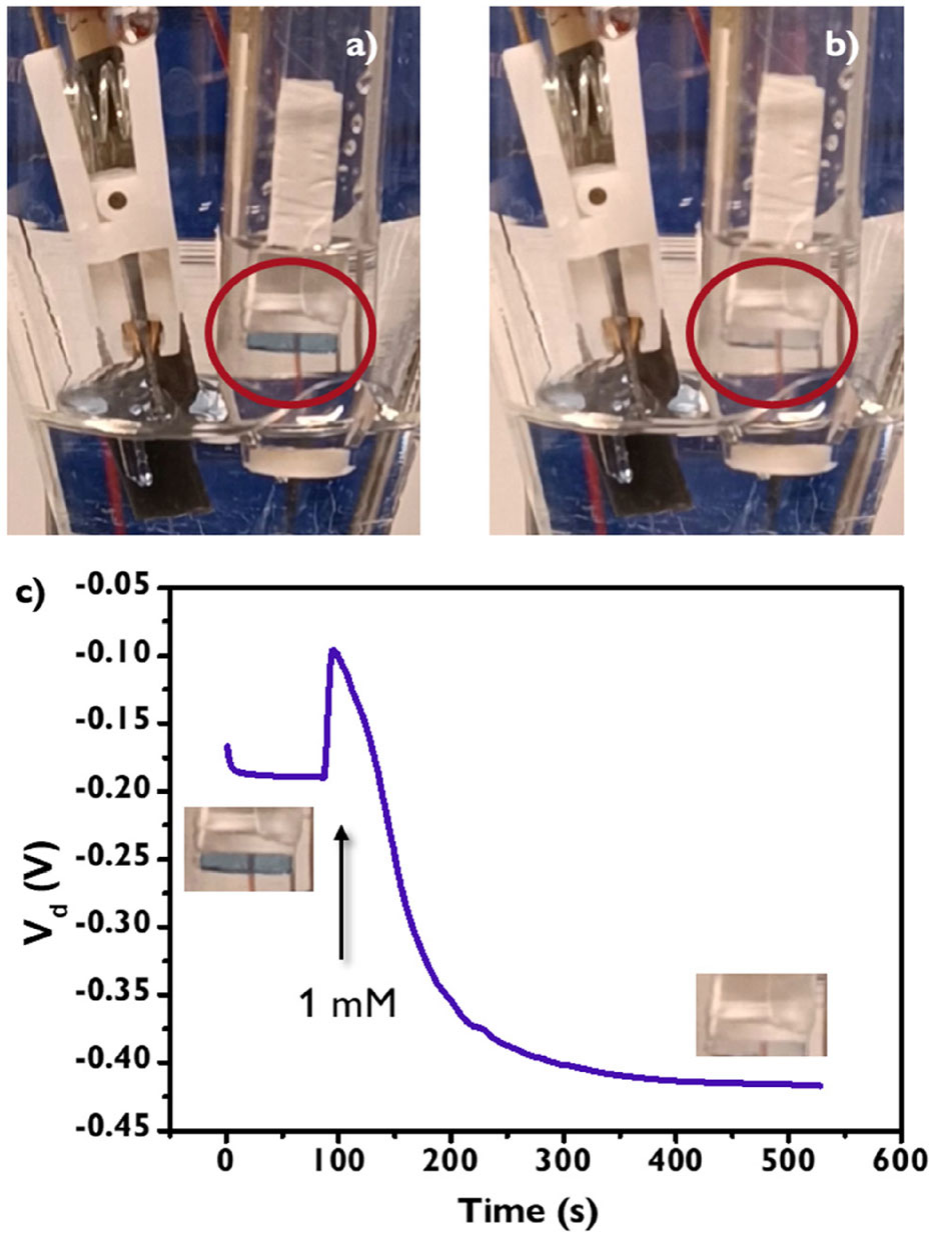
(a) Electrochemical cell with channel, GCE, and PB actuator in its oxidized state. (b) Electrochemical cell at *V*_g_ = +0.5 V and *I*_d_ = −50 µA after dopamine addition, with PB actuator in its reduced state. (c) *V*_d_ versus time plot, portraying the time of dopamine addition and relative actuator coloration.

Figure 5a,b shows the photos of dopamine‐driven system in the absence (Figure 5a) and in the presence (Figure 5b) of 1 mM dopamine in the electrolyte solution, with the complete video in Multimedia File 3. The system effectively controls the smart window actuator that exhibits a clear blue color in the dopamine‐free electrolyte solution and turns transparent after the dopamine addition. Figure 5c confirms the OECT operation by showing a clear negative *V*_d_ variation after the dopamine addition. Moreover, the blank experiment carried out by adding dopamine when all the circuits are open showed no color variation of the smart window actuator, thus confirming the role of OECT in controlling it. This protocol has proved robust, since various experiments varying *I*_d_ values were conducted, in accordance with the previous calibration curves. Regarding *I*_d_, the investigated values were −25, −50, and –100 µA, all of which provided the desired actuation (see Figures S15–S17 and Multimedia Files 4–6). Interestingly, for –25 and –50 µA measurements were performed with the same device confirming the measurement repeatability and durability of all the components of the electrochemical circuit. Contrarily, imposing a positive *I*_d_ as + 10 µA did not allow the achievement of the color change potential, in agreement with experiments described in previous section. Exploiting *E*_d_ range and values correlated to *I*_d_, as presented in Figure 3d, the right channel current could be selected depending on the actuator requirement.

To further demonstrate the processing and computing capabilities of the OECT, we used an ITO‐coated glass slide covered with polyaniline as the electrochromic actuator. This is possible due to polyaniline's ability to assume different oxidation states, thereby affecting its light absorption properties. Specifically, polyaniline can exist in several forms: the fully reduced leucoemeraldine form, which is typically colorless; the emeraldine form, which is green and can be further oxidized to the pernigraniline form, which appears blue‐violet. As shown in Figure S19b, these redox transitions occur at higher potentials compared to the redox processes of PB and therefore require a different approach to chemical signal processing.

**FIGURE 6:**
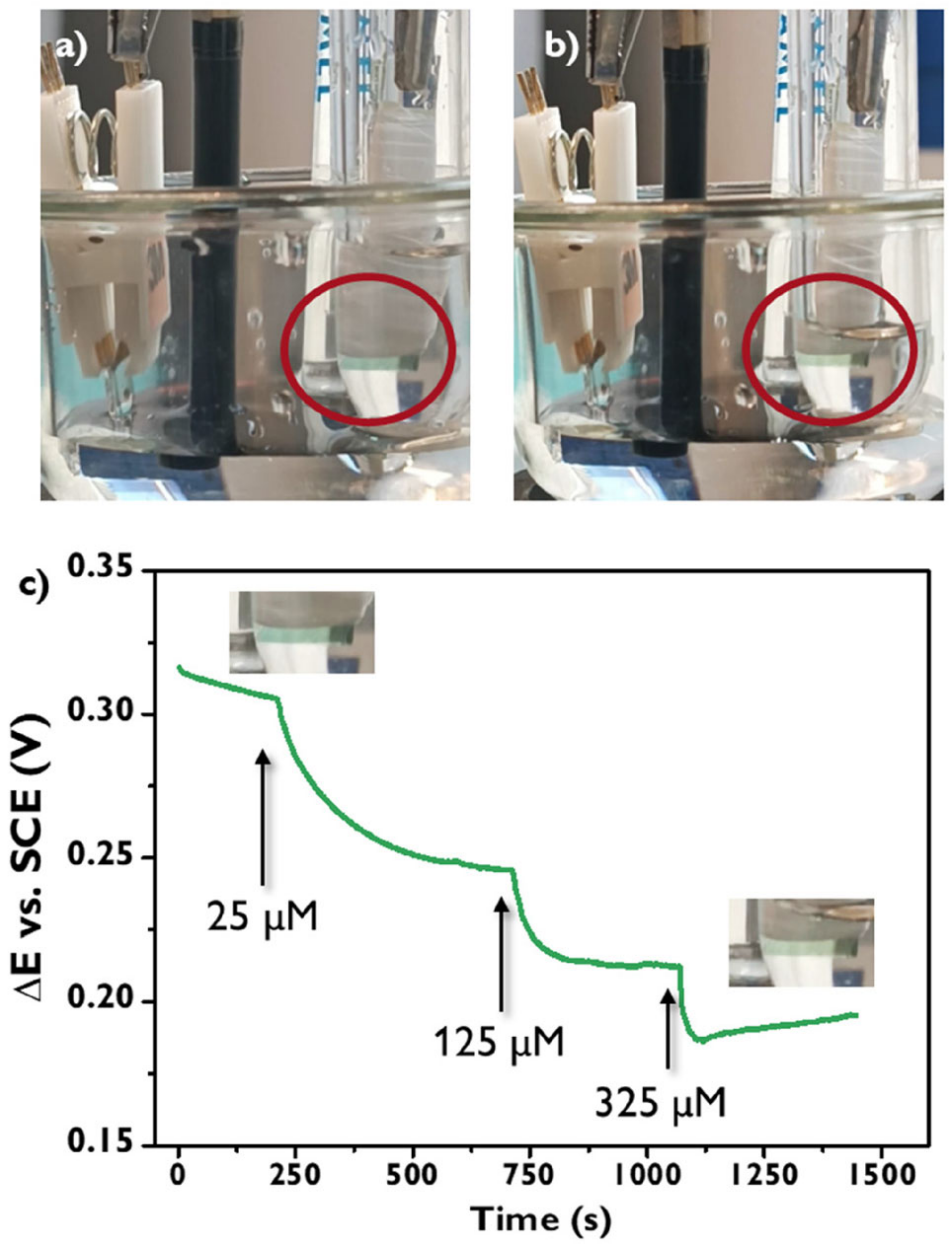
(a) Electrochemical cell with channel, GCE, and PANI actuator in its emeraldine state, inserted in a secondary cell with H_2_SO_4_ 0.1 M. (b) Electrochemical cell at *V*_g_ = +0.5 V and *I*_d_ = −50 µA after dopamine addition, with PANI actuator toward its leucoemeraldine state. (c) *E*_d_ versus time plot, portraying the time and concentration of dopamine addition and relative actuator coloration.

To achieve *E*_d_ variations at values greater than zero, we applied positive *I*_d_ values (+50 μA) while keeping *V*_g_ at 0.5 V. Under these conditions, changes in dopamine concentration resulted in a color shift of the actuator from green to pale green (Figure 6a,b, and Multimedia file 8). Furthermore, we observed that, even under these conditions, the system generates controlled *E*_d_ variations in response to different dopamine concentrations in the electrochemical cell, as shown in Figure 6c.

## 4 Conclusion

Bioelectronics has recently reached remarkable milestones in bridging biological and electronic systems, as well as in emulating complex biological processes, with far‐reaching implications for regenerative medicine and beyond. Although most signals exchanged within biological systems are chemical in nature, current technology predominantly operates through electrical stimuli. In this work, we present a novel strategy that seamlessly unites dopamine sensing, signal processing, and chemical actuation within a single, fully analog device, eliminating the need for digital processing altogether. By harnessing the unique properties of OECT technology, we achieved real‐time modulation of the drain potential, enabling precise control of electrochemical reactions triggered by dopamine detection.

Crucially, our approach required operating the OECT to produce a direct potential output. We optimized device operation by imposing a constant drain current, rather than the more commonly used constant drain voltage reported in the literature. Systematic characterization under various operating conditions provided deep insight into these potential variations and confirmed the feasibility of using OECTs for robust electrochemical actuation. Notably, we demonstrated that adjusting the gate voltage allows for fine modulation of the drain potential, making it possible to drive a range of electrochemical processes with high precision. Moreover, the transistor architecture can synergically combine the effects due to the conductivity variation of the channel and due to electrochemical reactions.

The capability of our device to reliably and repeatedly control an electrochemical reaction was showcased through the actuation of a PB‐based smart window. The ability to process and compute chemical signals in a defined, target output was ultimately demonstrated by controlling the electrochromic behavior of an ITO‐coated glass slide covered with polyaniline, an electrochromic material with an operating potential distinct from that of PB. This highlights the versatility of the technology, which can deliver different potential stimuli simply by modifying the sensing conditions, thus enabling adaptable and tunable responses for a variety of applications. These results pave the way for next‐generation integrated platforms that combine sensing, computation, and actuation in a single unit. Looking ahead, the versatility of OECT technology opens exciting opportunities for the development of neuromorphic functionalities and highly tunable bioelectronic actuators.

## Supporting Information

Additional supporting information can be found online in the Supporting Information section [57, 58, 59]. **Supporting Fig. S1:** a) Characteristic transfer curve of the OECT, with a velocity of 50 mV/s; b) characteristic output curves of the OECT at different V_g_ values (0 V ‐ 0.6 V) and V_d_ values (‐0.5 V – 0 V) with a velocity of 50 mV/s. **Supporting Fig. S2:** a) Cyclic voltammetry of a channel used for dopamine‐driven actuation, conducted in KCl 0.1 M between ‐0.2 V and +0.6 V vs. SCE at 50 mV/s, with GCE as working electrode and a Pt wire as counter electrode; b) cyclic voltammetry of a PB‐ITO electrode of 0.1 cm^2^ of area, conducted in KCl 0.1 M between ‐0.2 and +0.4 V vs. SCE at 50 mV/s, with a similar set‐up. **Supporting Fig. S3:** a) V_d_ vs time plot obtained with V_g_ = 0.3 V and I_d_ = ‐10 µA with dopamine additions in the cell. c) ΔV_d_ vs. Da concentration plot. d) E_d_ vs. time plot obtained in the same moment of b). e) ΔE_d_ vs. DA concentration plot. **Supporting Fig. S4:** a) V_d_ vs time plot obtained with V_g_ = 0.4 V and I_d_ = ‐10 µA with dopamine additions in the cell. c) ΔV_d_ vs. DA concentration plot. d) E_d_ vs. time plot obtained in the same moment of b). e) ΔE_d_ vs. DA concentration plot. **Supporting Fig. S5:** a) V_d_ vs time plot obtained with V_g_ = 0.5 V and I_d_ = ‐10 µA with dopamine additions in the cell. c) ΔV_d_ vs. DA concentration plot. d) E_d_ vs. time plot obtained in the same moment of b). e) ΔE_d_ vs. DA concentration plot. **Supporting Fig. S6:** a) V_d_ vs time plot obtained with V_g_ = 0.6 V and I_d_ = ‐10 µA with dopamine additions in the cell. c) ΔV_d_ vs. DA concentration plot. d) E_d_ vs. time plot obtained in the same moment of b). e) ΔE_d_ vs. DA concentration plot. **Supporting Fig. S7:** a) V_d_ vs time plot obtained with V_g_ = 0.5 V and I_d_ = ‐50 µA with dopamine additions in the cell. c) ΔV_d_ vs. DA concentration plot. d) E_d_ vs. time plot obtained in the same moment of b). e) ΔE_d_ vs. DA concentration plot. **Supporting Fig. S8:** a) V_d_ vs time plot obtained with V_g_ = 0.5 V and I_d_ = ‐25 µA with dopamine additions in the cell. c) ΔV_d_ vs. DA concentration plot. d) E_d_ vs. time plot obtained in the same moment of b). e) ΔE_d_ vs. DA concentration plot. **Supporting Fig. S9:** a) V_d_ vs time plot obtained with V_g_ = 0.5 V and I_d_ = ‐10 µA with dopamine additions in the cell. c) ΔV_d_ vs. DA concentration plot. d) E_d_ vs. time plot obtained in the same moment of b). e) ΔE_d_ vs. DA concentration plot. **Supporting Fig. S10:** a) V_d_ vs time plot obtained with V_g_ = 0.5 V and I_d_ = 0 µA with dopamine additions in the cell. c) ΔV_d_ vs. DA concentration plot. d) E_d_ vs. time plot obtained in the same moment of b). e) ΔE_d_ vs. DA concentration plot. **Supporting Fig. S11:** a) V_d_ vs time plot obtained with V_g_ = 0.5 V and I_d_ = +10 µA with dopamine additions in the cell. c) ΔV_d_ vs. DA concentration plot. d) E_d_ vs. time plot obtained in the same moment of b). e) ΔE_d_ vs. DA concentration plot. **Supporting Fig. S12:** a) V_d_ vs time plot obtained with V_g_ = 0.5 V and I_d_ = +25 µA with dopamine additions in the cell. c) ΔV_d_ vs. DA concentration plot. d) E_d_ vs. time plot obtained in the same moment of b). e) ΔE_d_ vs. DA concentration plot. **Supporting Fig. S13:** a) V_d_ vs time plot obtained with V_g_ = 0.5 V and I_d_ = +50 µA with dopamine additions in the cell. c) ΔV_d_ vs. DA concentration plot. d) E_d_ vs. time plot obtained in the same moment of b). e) ΔE_d_ vs. DA concentration plot. **Supporting Fig. S14:** Calibrations curves with error bars repeated thrice with V_g_ = +0.5 V: a) E_d_ vs. DA concentration plot at I_d_ = ‐25 µA; b) E_d_ vs. DA concentration plot at I_d_ = ‐50 µA; c) V_d_ vs. DA concentration plot at I_d_ = ‐25 µA obtained at the same time as a); d) V_d_ vs. DA concentration plot at I_d_ = ‐50 µA obtained at the same time as b). **Supporting Fig. S15:** V_d_ vs. time plot obtained from the dopamine‐driven electrochromic actuation with an I_d_ equal to ‐25 µA in PBS 0.1 M pH 7 with dopamine additions, shown in Multimedia file 4). **Supporting Fig. S16:** V_d_ vs. time plot obtained from the dopamine‐driven electrochromic actuation with an I_d_ equal to ‐100 µA in PBS 0.1 M pH 7 with dopamine additions, shown in Multimedia file 5). **Supporting Fig. S17:** V_d_ vs. time plot obtained from the dopamine‐driven electrochromic actuation with an I_d_ equal to ‐50 µA and no GCE as functioning gate electrode in PBS 0.1 M pH 7 with dopamine additions, shown in Multimedia file 6). **Supporting Fig. S18:** V_d_ vs. time plot obtained from the dopamine‐driven electrochromic actuation with an I_d_ equal to ‐50 µA performed by a different operator (Multimedia file 7). **Supporting Fig. S19:** a) Electrochemical deposition of PANI through cyclic voltammetry from ‐0.2 to +1.0 V vs. SCE, with the resulting currents expressed over ITO glass area; b) absorption spectrum of PANI deposited on ITO glass at +0.8 V (purple), +0.4 V (green), 0 V (pale green) and ‐0.2 V (grey). **Supporting Table S1:** Slope values and R^2^ of calibration curves obtained at various V_g_ with a fixed I_d_ equal to ‐10 µA. **Supporting Table S2:** Slope values and R^2^ of calibration curves obtained at various I_d_ with a fixed V_g_ equal to +0.5 V. **Supporting Table S3:** a) LOD of calibration curves obtained at various I_d_, b) at various V_g_.

## Author Contributions

**Giada D’Altri:** conceptualization (supporting), data curation (lead), formal analysis (lead), investigation (lead), methodology (supporting), writing – original draft (lead), writing – review and editing (equal). **Federica Mariani:** conceptualization (supporting), methodology (supporting), supervision (equal), writing – review and editing (equal). **Filippo Bonafè**: conceptualization (supporting), investigation (supporting), writing – review and editing (equal). **Francesco Decataldo:** conceptualization (supporting), writing – review and editing (equal). **Marta Tessarolo:** conceptualization (supporting), supervision (supporting), writing – review and editing (equal). **Beatrice Fraboni:** conceptualization (supporting), funding acquisition (lead), resources (lead), supervision (supporting), writing – review and editing (equal). **Erika Scavetta:** conceptualization (supporting), funding acquisition (lead), resources (lead), supervision (supporting), writing – original draft (supporting), writing – review and editing (equal). **Isacco Guanlandi:** conceptualization (lead), formal analysis (supporting), funding acquisition (equal), methodology (lead), resources (equal), supervision (lead), writing – original draft (supporting), writing – review and editing (equal).

## 6 Conflicts of Interest

The authors declare no conflicts of interest.

## Supporting information

Supplementary Material

## Data Availability

The data that support the findings of this study are available in the supplementary material of this article.
