# Supplementary material for: Dopamine‐Mediated Analog Control of Electrochromic Reactions Through Organic Electrochemical Transistor
Source: Small Sci. 2026 Mar 17;6(3):e202500635. doi: 10.1002/smsc.202500635 (PMC13097497; doi:10.1002/smsc.202500635)
Supplement: Supplementary file 1 — Supplementary Material [file SMSC-6-e202500635-s001.pdf]

Supplementary information

Dopamine-mediated analog control of electrochromic reactions through organic electrochemical transistor

Giada D'Altri[a], Federica Mariani[a], Filippo Bonafè[b], Francesco Decataldo[c], Marta Tessarolo[b], Beatrice Fraboni[b], Erika Scavetta[a], Isacco Gualandi[a]*

[a] *Department of Industrial Chemistry "Toso Montanari", University of Bologna, Via Piero Gobetti, 85, Bologna, 40129, Italy*

[b] *Department of Physics and Astronomy "Augusto Righi", University of Bologna, Viale Berti Pichat 6/2, Bologna, 40127, Italy*

[c] *Department of Medical and Surgical Sciences, University of Bologna, Via Pelagio Palagi, 9, Bologna 40138, Italy*

List of contents

Fig. SI 1	Characteristic transfer and output curves of OECT
Fig. S2	Electrochemical characterization of PEDOT:PSS channel and PB-ITO actuator
Fig. SI 3	V_d and E_d vs. time and concentrations plots, with V_g = +0.3 V and I_d = -10 µA
Fig. SI 4	V_d and E_d vs. time and concentrations plots, with V_g = +0.4 V and I_d = -10 µA
Fig. SI 5	V_d and E_d vs. time and concentrations plots, with V_g = +0.5 V and I_d = -10 µA
Fig. SI 6	V_d and E_d vs. time and concentrations plots, with V_g = +0.6 V and I_d = -10 µA
Fig. SI 7	V_d and E_d vs. time and concentrations plots, with V_g = +0.5 V and I_d = -50 µA
Fig. SI 8	V_d and E_d vs. time and concentrations plots, with V_g = +0.5 V and I_d = -25 µA
Fig. SI 9	V_d and E_d vs. time and concentrations plots, with V_g = +0.5 V and I_d = -10 µA
Fig. SI 10	V_d and E_d vs. time and concentrations plots, with V_g = +0.5 V and I_d = 0 µA
Fig. SI 11	V_d and E_d vs. time and concentrations plots, with V_g = +0.5 V and I_d = +10 µA
Fig. SI 12	V_d and E_d vs. time and concentrations plots, with V_g = +0.5 V and I_d = +25 µA
Fig. SI 13	V_d and E_d vs. time and concentrations plots, with V_g = +0.5 V and I_d = +50 µA
Fig. SI 14	V_d and E_d vs. DA concentrations plots with error bars with Vg = +0.5 V and Id = -25, -50 µA
Tab. SI 1	Slope values and R^2 of calibration curves at various V_g
Tab. SI 2	Slope values and R^2 of calibration curves at various I_d
Tab. SI 3	LOD of calibration curves at various I_d and various V_g
Fig. SI 15	V_d vs. time plot during actuation experiment (I_d = -25 µA)
Fig. SI 16	V_d vs. time plot during actuation experiment (I_d = -100 µA)
Fig. SI 17	V_d vs. time plot during actuation experiment (I_d = -50 µA) with no operating gate
Fig. SI 18	V_d vs. time plot during actuation experiment (I_d = -50 µA) performed by a different operator
Fig. SI 19	Electrochemical PANI deposition and spectroelectrochemical characterization
SI 1	Preparation and characterization of PANI-based ITO actuator
SI 2	List of Multimedia contents

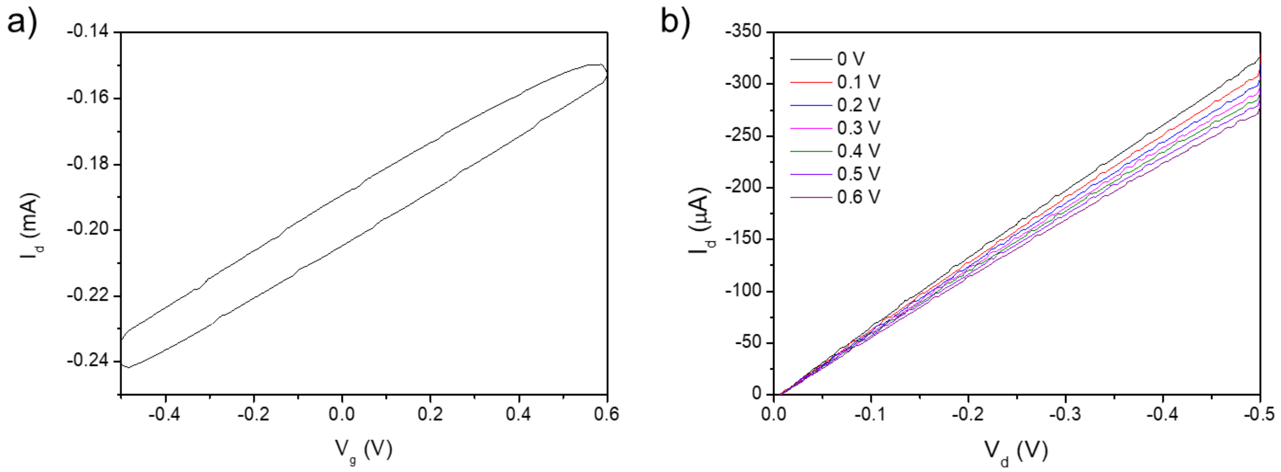

Figure SI 1. a) Characteristic transfer curve of the OECT, with a velocity of 50 mV/s; b) characteristic output curves of the OECT at different V_g values (0 V - 0.6 V) and V_d values (-0.5 V – 0 V) with a velocity of 50 mV/s.

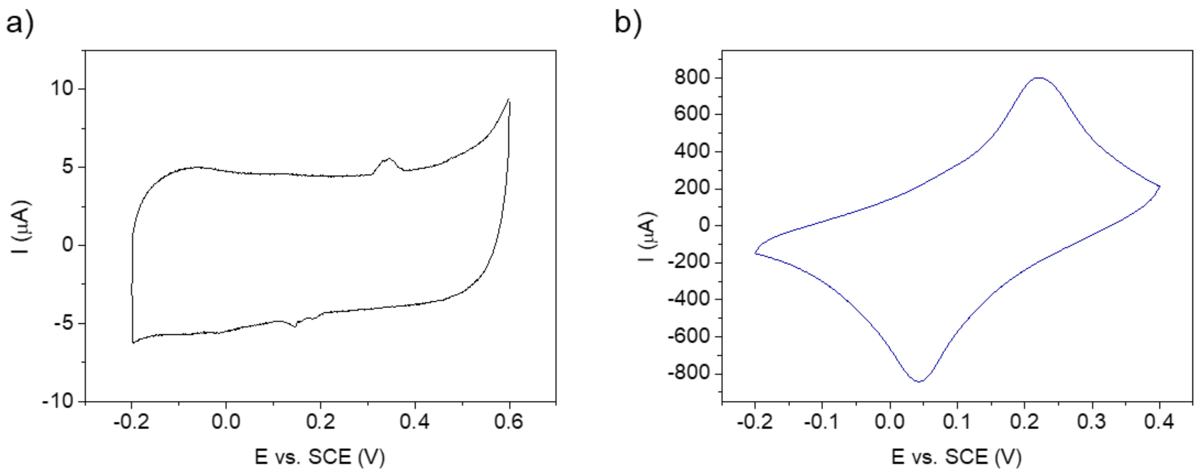

Fig. SI 2 a) Cyclic voltammetry of a channel used for dopamine-driven actuation, conducted in KCl 0.1 M between -0.2 V and +0.6 V vs. SCE at 50 mV/s, with GCE as working electrode and a Pt wire as counter electrode; b) cyclic voltammetry of a PB-ITO electrode of 0.1 cm^2 of area, conducted in KCl 0.1 M between -0.2 and +0.4 V vs. SCE at 50 mV/s, with a similar set-up.

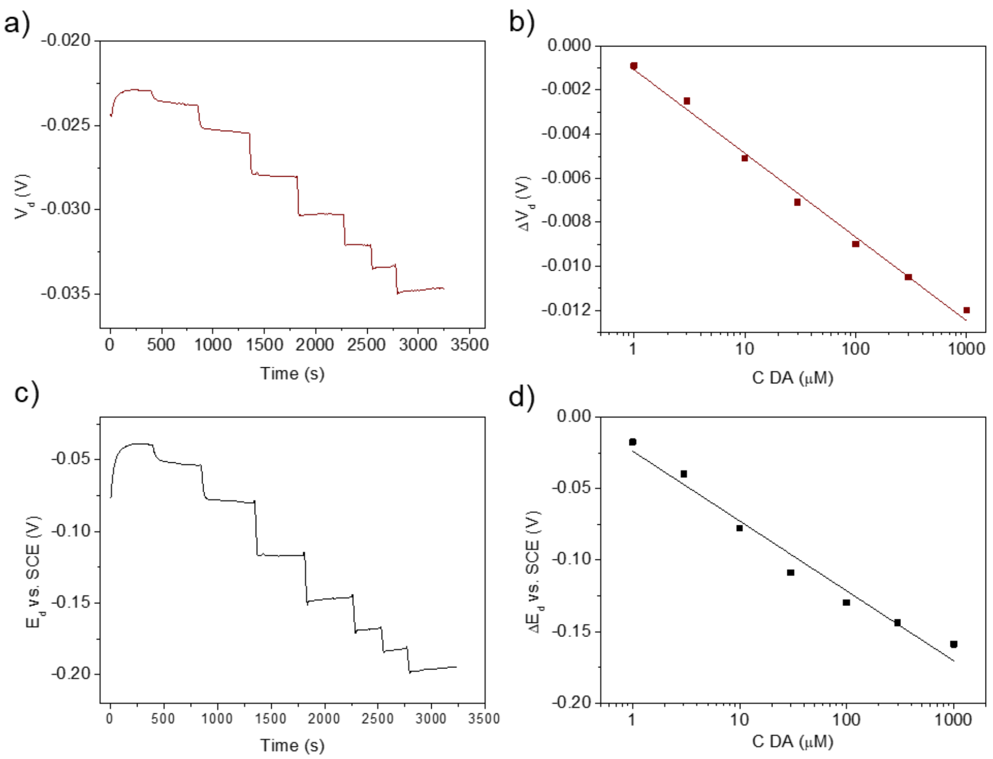

Fig. SI 3 a) V_d vs time plot obtained with V_g = 0.3 V and I_d = -10 μA with dopamine additions in the cell. c) ΔV_d vs. Da concentration plot. d) E_d vs. time plot obtained in the same moment of b). e) ΔE_d vs. DA concentration plot.

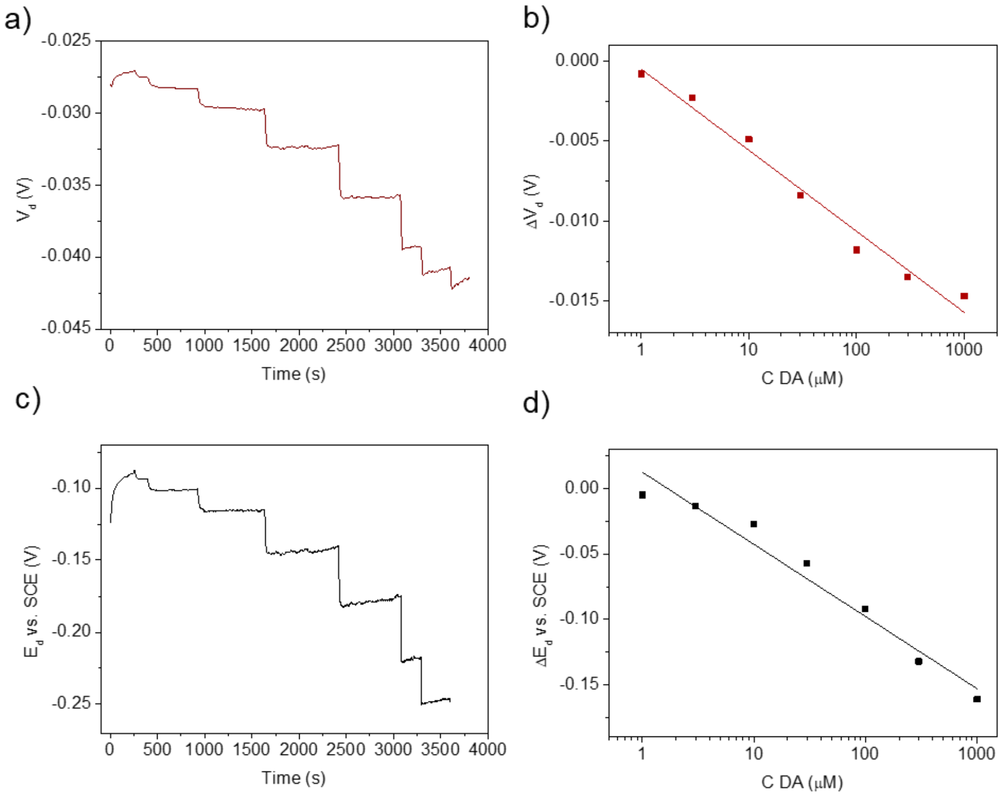

Fig. SI 4 a) V_d vs time plot obtained with V_g = 0.4 V and I_d = -10 μA with dopamine additions in the cell. c) ΔV_d vs. DA concentration plot. d) E_d vs. time plot obtained in the same moment of b). e) ΔE_d vs. DA concentration plot.

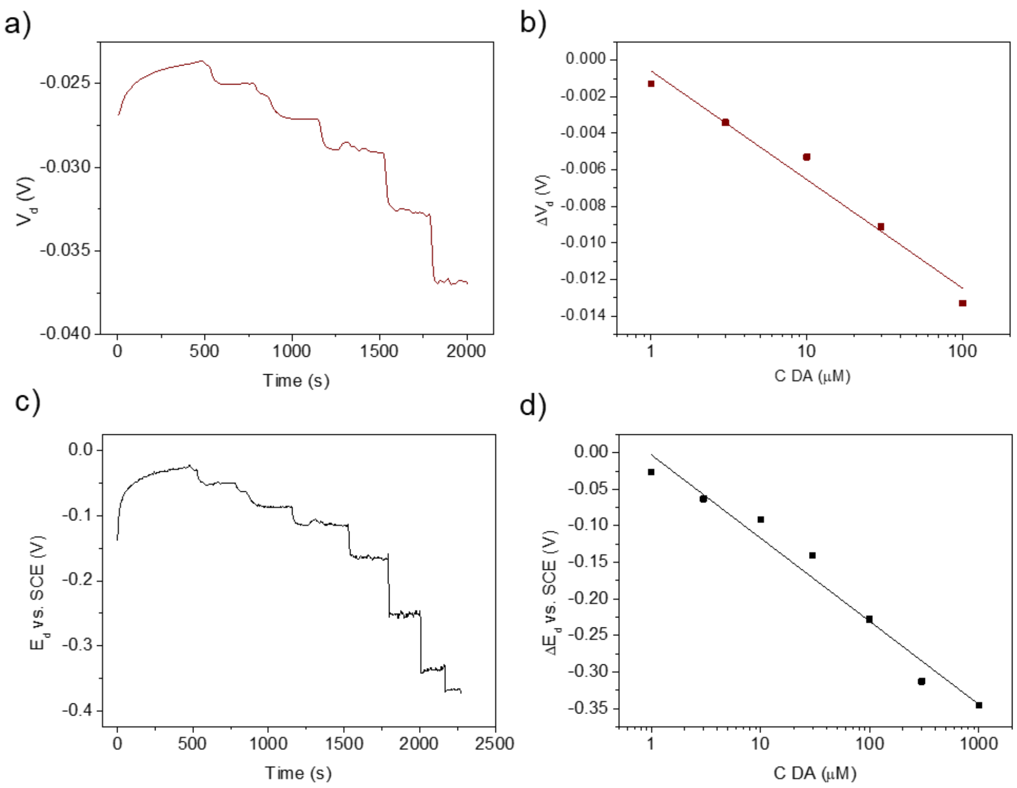

Fig. SI 5 a) V_d vs time plot obtained with V_g = 0.5 V and I_d = -10 µA with dopamine additions in the cell. c) ΔV_d vs. DA concentration plot. d) E_d vs. time plot obtained in the same moment of b). e) ΔE_d vs. DA concentration plot.

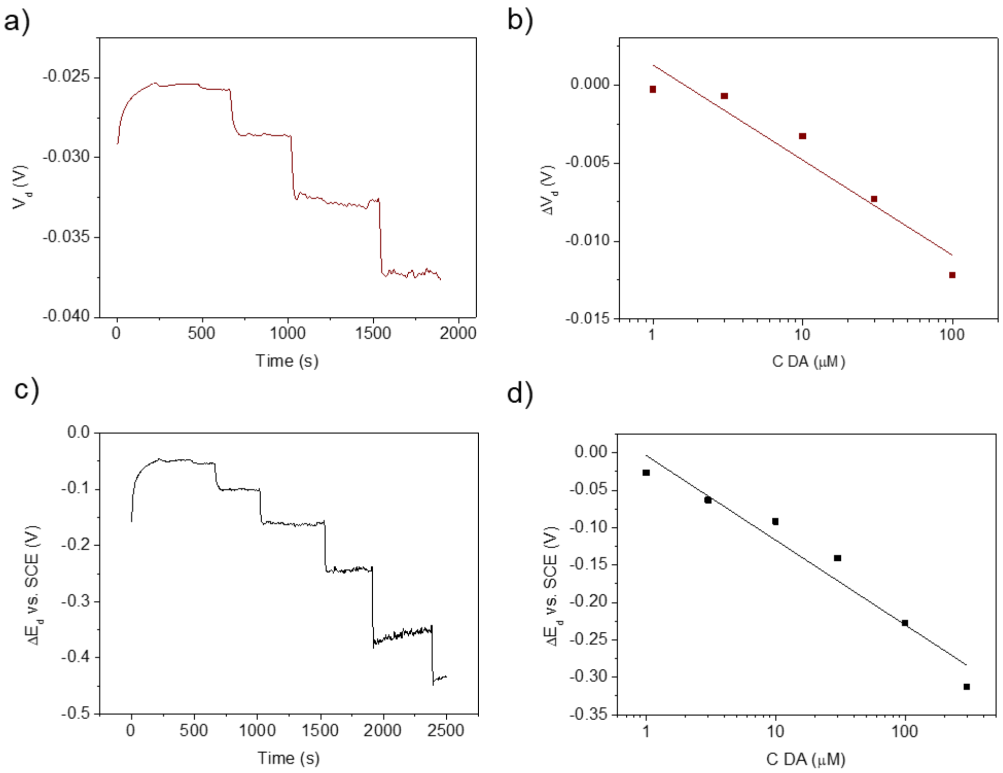

Fig. SI 6 a) V_d vs time plot obtained with V_g = 0.6 V and I_d = -10 µA with dopamine additions in the cell. c) ΔV_d vs. DA concentration plot. d) E_d vs. time plot obtained in the same moment of b). e) ΔE_d vs. DA concentration plot.

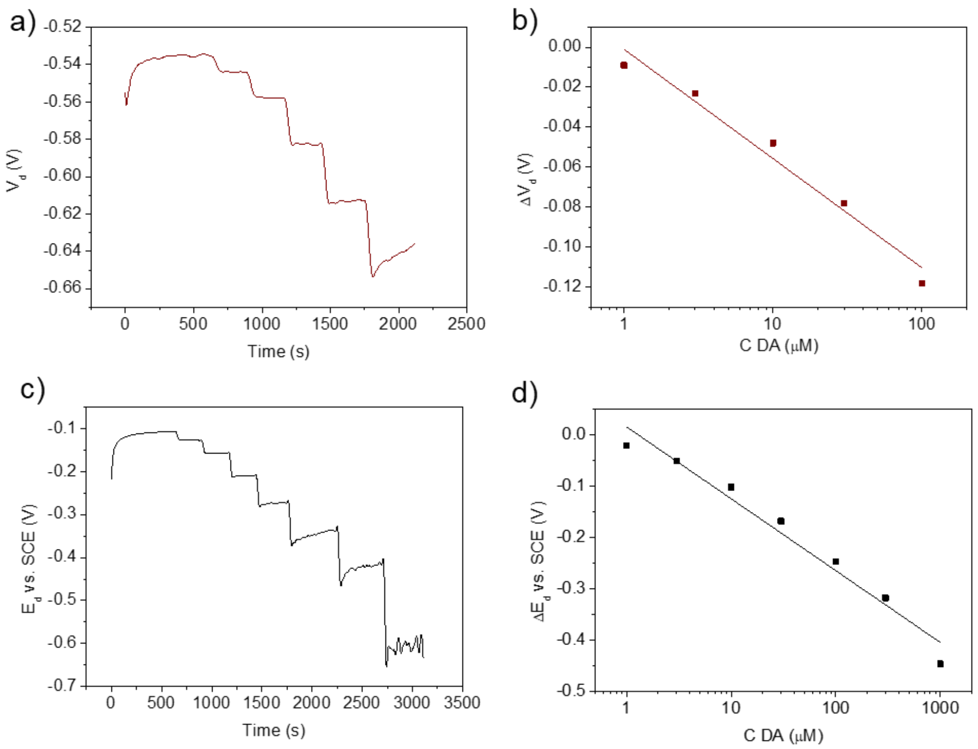

Fig. SI 7 a) V_d vs time plot obtained with V_g = 0.5 V and I_d = -50 µA with dopamine additions in the cell. c) ΔV_d vs. DA concentration plot. d) E_d vs. time plot obtained in the same moment of b). e) ΔE_d vs. DA concentration plot.

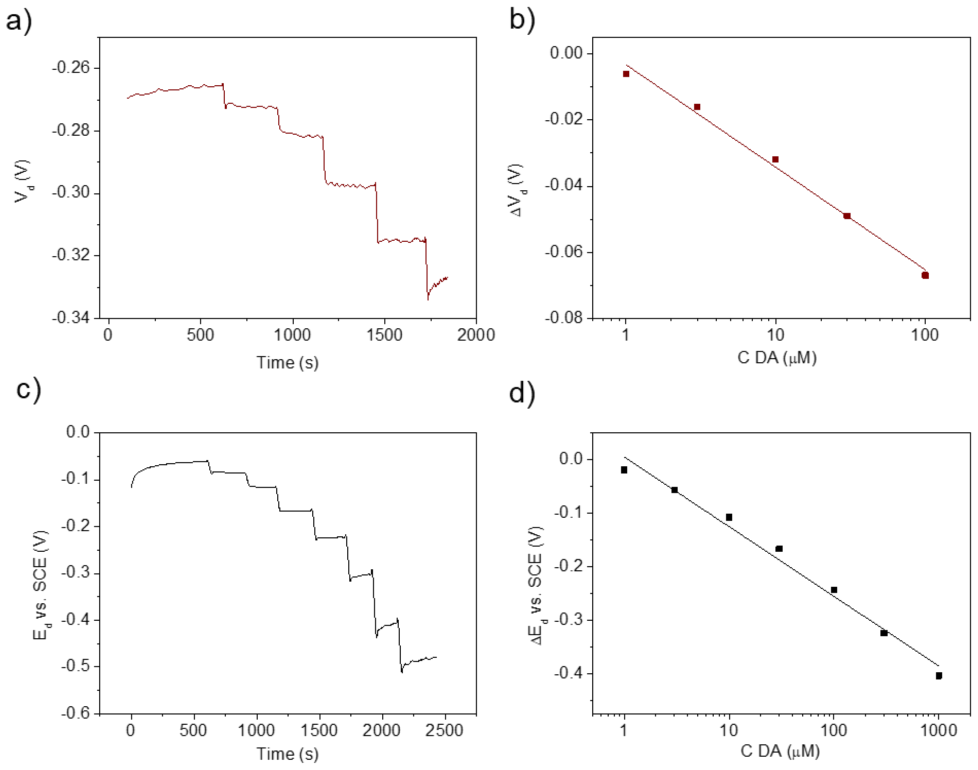

Fig. SI 8 a) V_d vs time plot obtained with V_g = 0.5 V and I_d = -25 µA with dopamine additions in the cell. c) ΔV_d vs. DA concentration plot. d) E_d vs. time plot obtained in the same moment of b). e) ΔE_d vs. DA concentration plot.

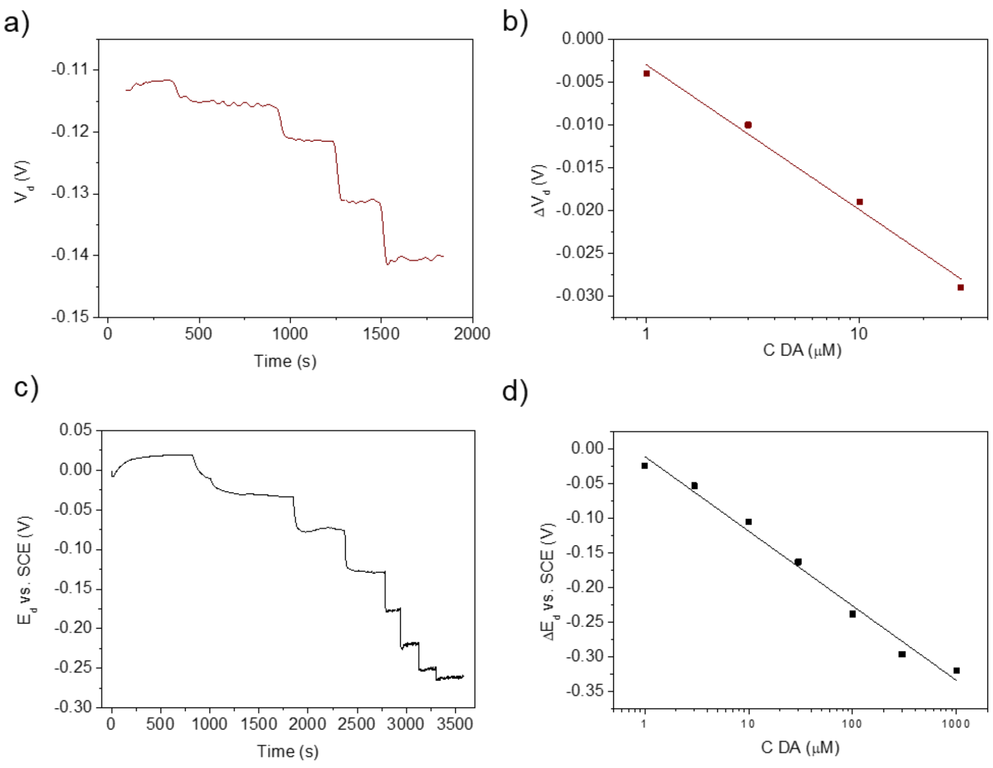

Fig. SI 9 a) V_d vs time plot obtained with V_g = 0.5 V and I_d = -10 μA with dopamine additions in the cell. c) ΔV_d vs. DA concentration plot. d) E_d vs. time plot obtained in the same moment of b). e) ΔE_d vs. DA concentration plot.

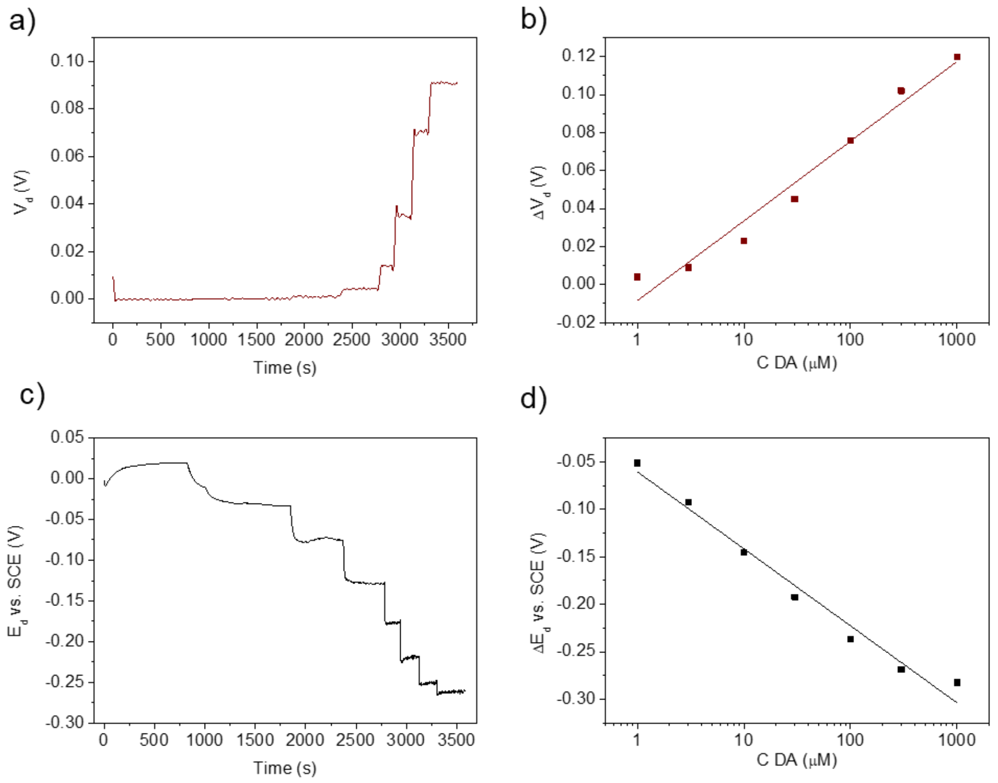

Fig. SI 10 a) V_d vs time plot obtained with V_g = 0.5 V and I_d = 0 μA with dopamine additions in the cell. c) ΔV_d vs. DA concentration plot. d) E_d vs. time plot obtained in the same moment of b). e) ΔE_d vs. DA concentration plot.

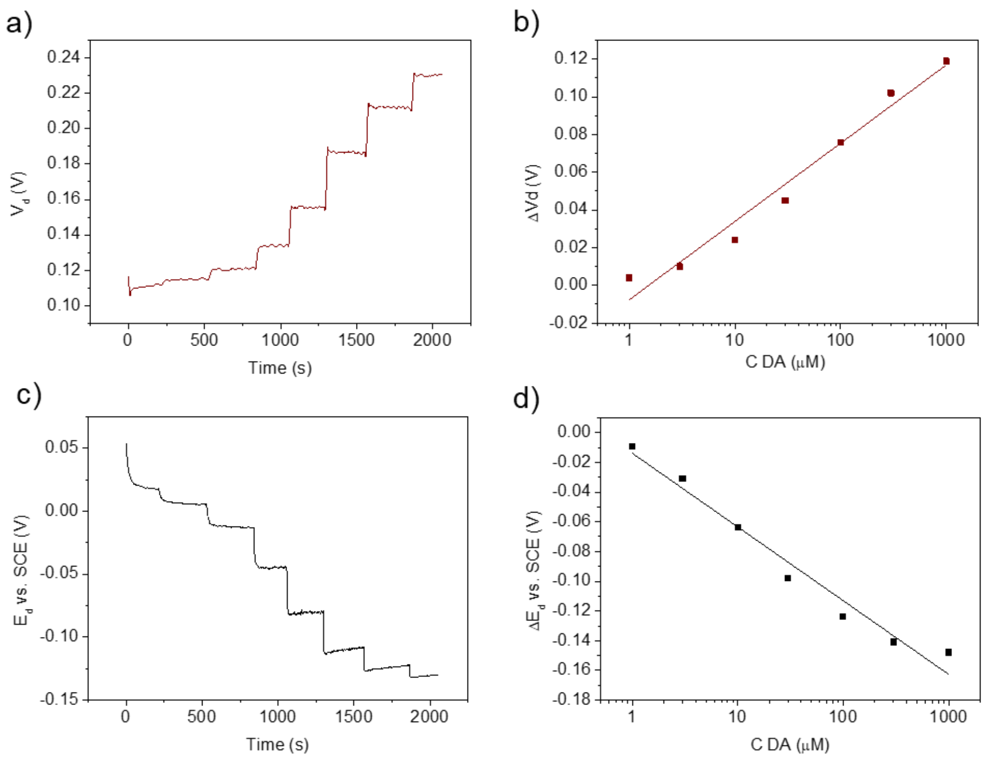

Fig. SI 11 a) V_d vs time plot obtained with $V_g = 0.5$ V and $I_d = +10$ µA with dopamine additions in the cell. c) ΔV_d vs. DA concentration plot. d) E_d vs. time plot obtained in the same moment of b). e) ΔE_d vs. DA concentration plot.

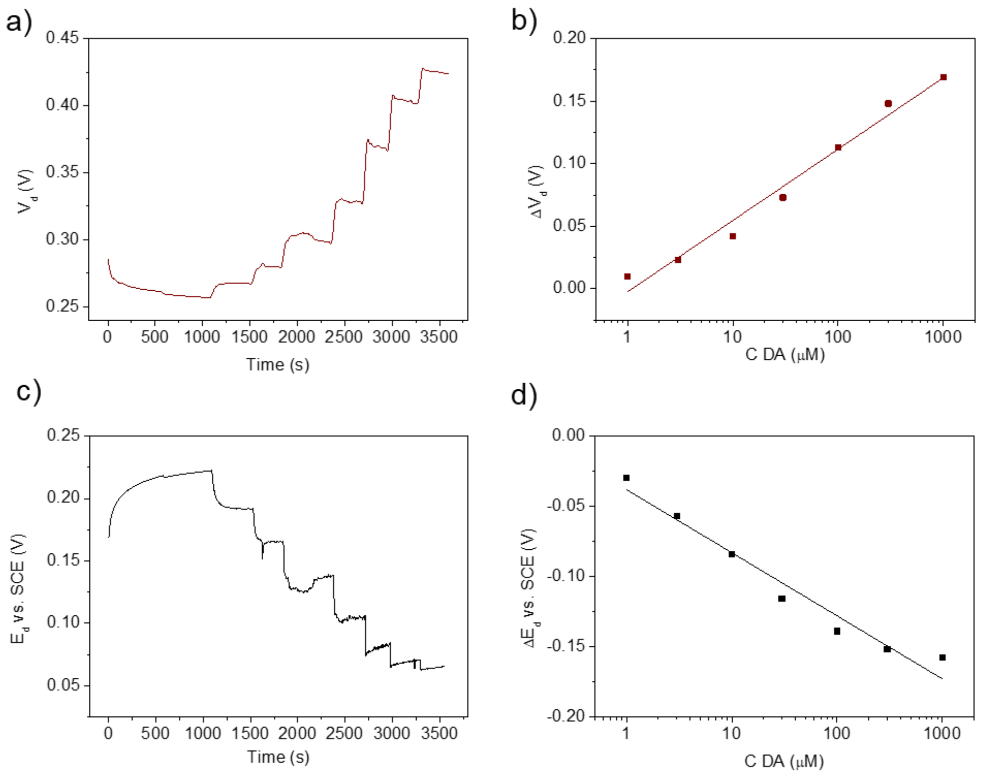

Fig. SI 12 a) V_d vs time plot obtained with $V_g = 0.5$ V and $I_d = +25$ µA with dopamine additions in the cell. c) ΔV_d vs. DA concentration plot. d) E_d vs. time plot obtained in the same moment of b). e) ΔE_d vs. DA concentration plot.

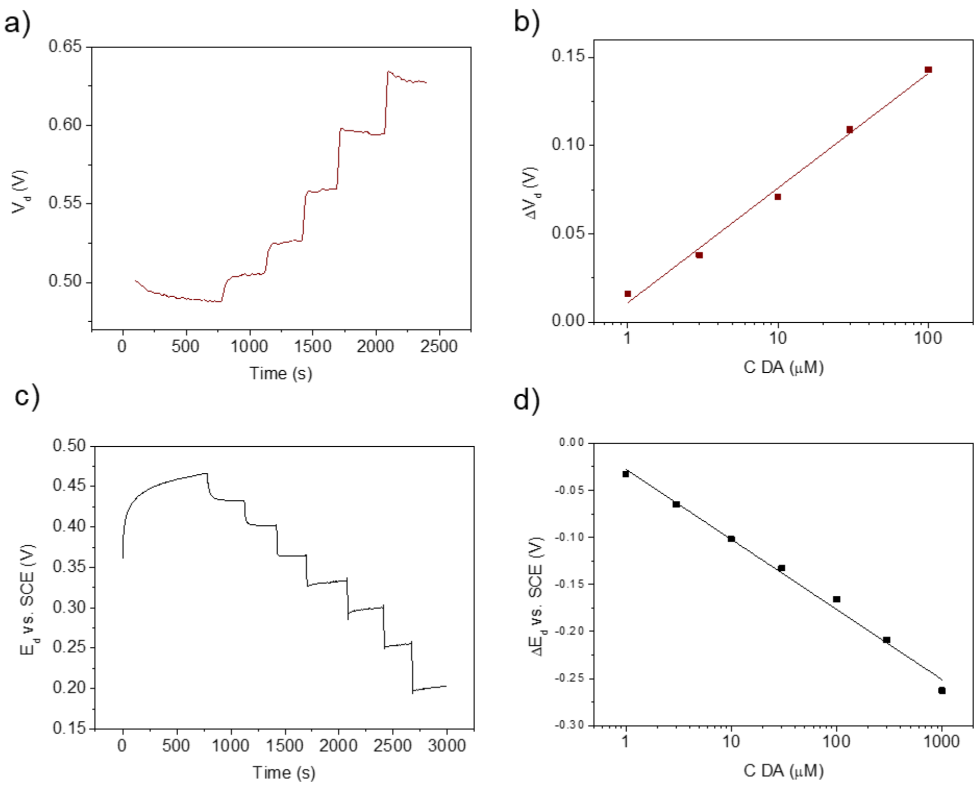

Fig. SI 13 a) V_d vs time plot obtained with $V_g = 0.5$ V and $I_d = +50$ μA with dopamine additions in the cell. c) ΔV_d vs. DA concentration plot. d) E_d vs. time plot obtained in the same moment of b). e) ΔE_d vs. DA concentration plot.

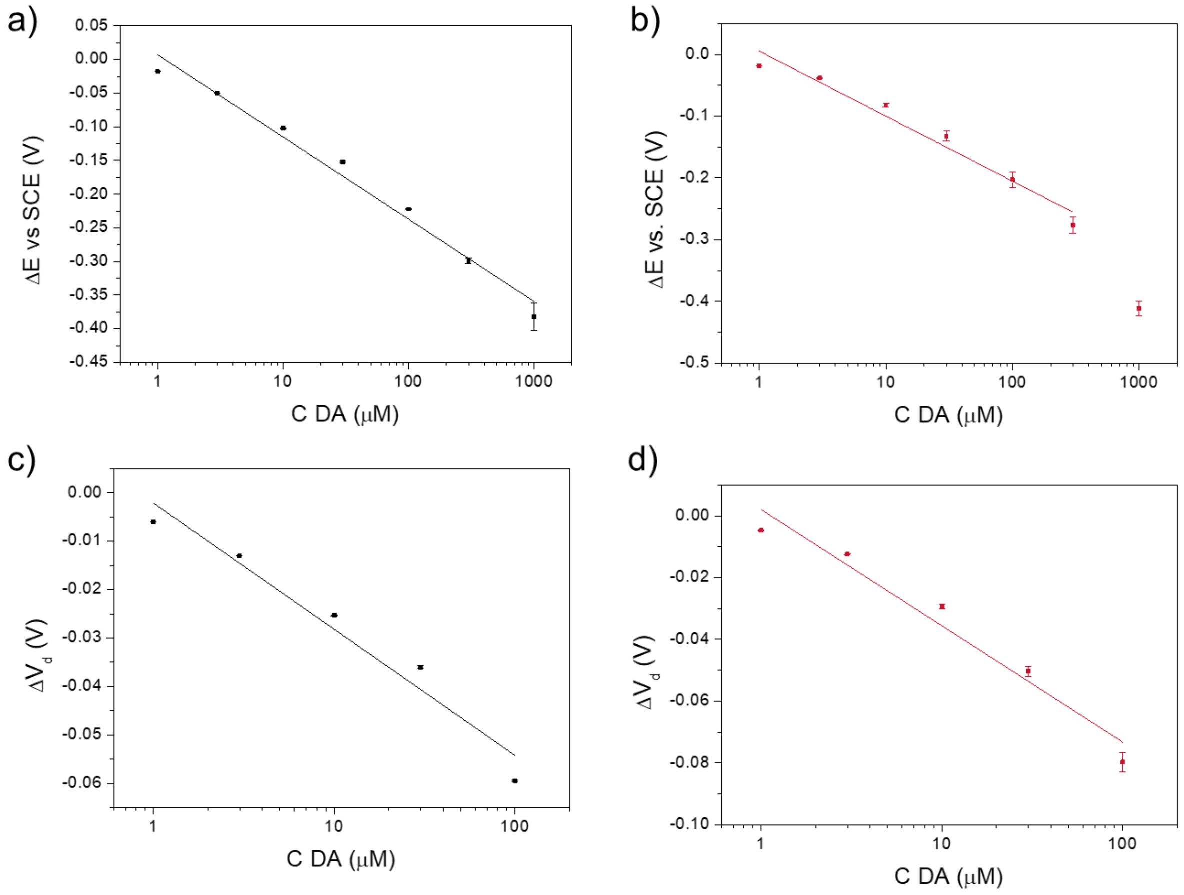

Figure SI 14. Calibrations curves with error bars repeated thrice with V_g = +0.5 V: a) E_d vs. DA concentration plot at I_d = -25 µA; b) E_d vs. DA concentration plot at I_d = -50 µA; c) V_d vs. DA concentration plot at I_d = -25 µA obtained at the same time as a); d) V_d vs. DA concentration plot at I_d = -50 µA obtained at the same time as b).

Tab. SI 1) Slope values and R^2 of calibration curves obtained at various V_g with a fixed I_d equal to -10 µA.

V_d			E_d		
V_g (V)	Slope (mV per decade)	R^2	V_g (V)	Slope (mV per decade)	R^2
0.2	-0.00213	0.998	0.2	-0.033	0.988
0.3	-0.0038	0.991	0.3	-0.049	0.966
0.4	-0.0051	0.976	0.4	-0.062	0.979
0.5	-0.0059	0.959	0.5	-0.102	0.936
0.6	-0.00763	0.972	0.6	-0.127	0.942

Tab. SI 2) Slope values and R^2 of calibration curves obtained at various I_d with a fixed V_g equal to +0.5 V.

V_d			E_d		
I_d (µA)	Slope (mV per decade)	R^2	I_d (µA)	Slope (mV per decade)	R^2
-50	-0.055	0.963	-50	-0.14	0.961
-25	-0.031	0.989	-25	-0.13	0.982
-10	-0.017	0.983	-10	-0.108	0.983
0	0.042	0.963	0	-0.081	0.975

10	0.042	0.965	10	-0.05	0.964
25	0.057	0.975	25	-0.051	0.985
50	0.065	0.991	50	-0.07	0.99

Tab. SI 3 a) LOD of calibration curves obtained at various I_d, b) at various V_g.

a)

I_d (μA)	LOD V_d (μM)	LOD E_d (μM)
-50	0.229	0.341
-25	0.249	0.439
-10	0.25	0.425
0	0.635	0.518
10	0.617	0.621
25	0.516	0.705
50	0.461	0.647

b)

V_g (V)	LOD V_d (μM)	LOD E_d (μM)
0.2	0.123	0.36
0.3	0.302	0.605
0.4	0.45	0.709
0.5	0.486	0.615
0.6	0.779	0.807

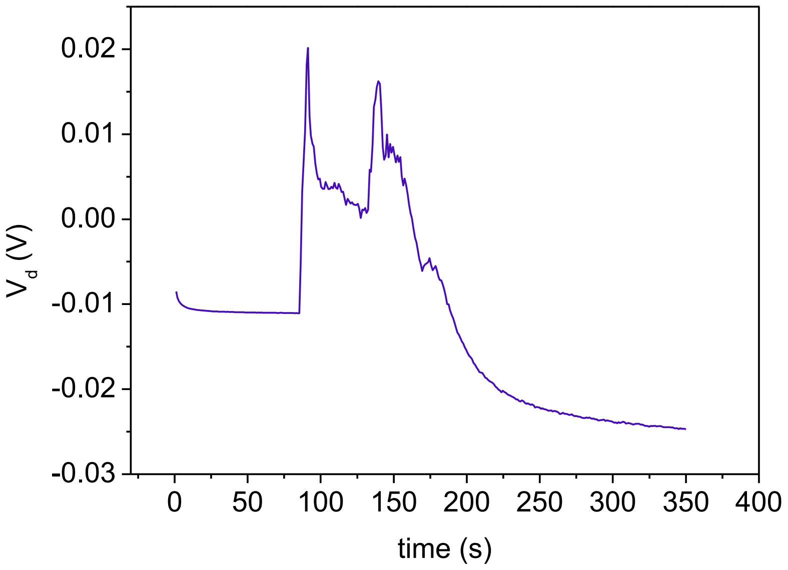

Fig. SI 15) V_d vs. time plot obtained from the dopamine-driven electrochromic actuation with an I_d equal to -25 μA in PBS 0.1 M pH 7 with dopamine additions, shown in Multimedia file 4).

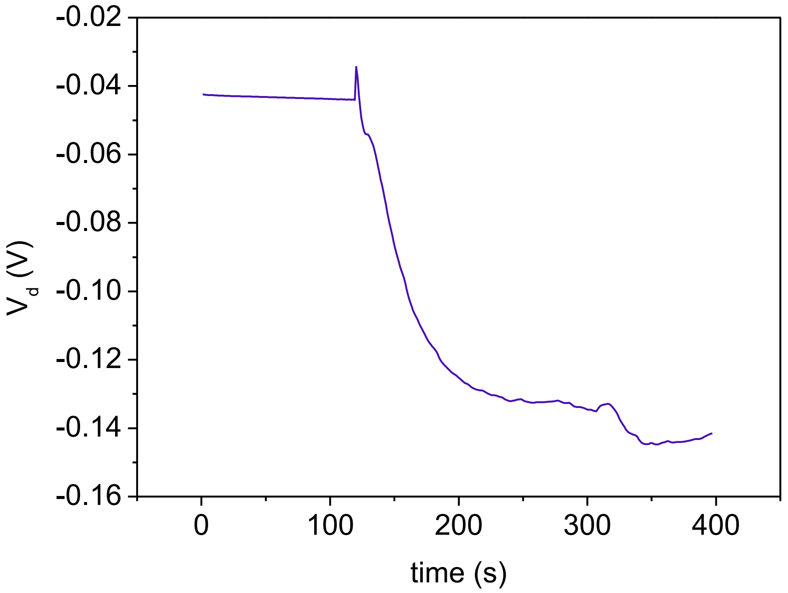

Fig. SI 16) V_d vs. time plot obtained from the dopamine-driven electrochromic actuation with an I_d equal to -100 μA in PBS 0.1 M pH 7 with dopamine additions, shown in Multimedia file 5).

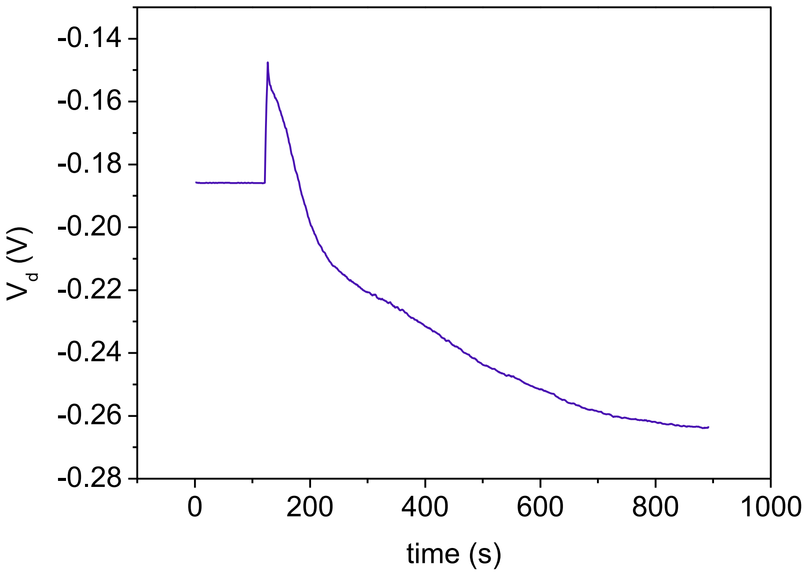

Fig. SI 17) V_d vs. time plot obtained from the dopamine-driven electrochromic actuation with an I_d equal to -50 μA and no GCE as functioning gate electrode in PBS 0.1 M pH 7 with dopamine additions, shown in Multimedia file 6).

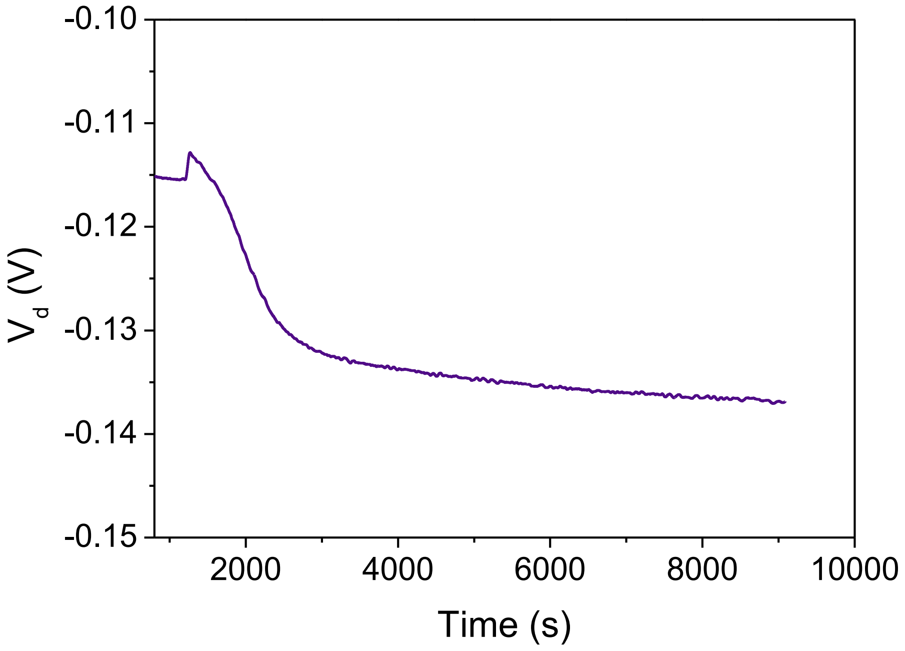

Fig. SI 18) V_d vs. time plot obtained from the dopamine-driven electrochromic actuation with an I_d equal to -50 µA performed by a different operator (Multimedia file 7).

SI 1 - Relationship between E_d and V_d

The E_d and V_d values are intrinsically related because they both describe the electrical potential of the drain electrode. On the one hand, V_d is defined as the potential of the drain with respect to the source terminal, which is not referenced to an electrochemical system with a fixed potential. On the other hand, E_d is measured with respect to a saturated calomel electrode, which provides a stable electrochemical reference potential when an ionic circuit is present. In addition, E_d is determined under open-circuit conditions, so that no faradaic processes occur at the reference electrode, thereby avoiding any perturbation of its electrochemical potential. Under these conditions, the measured potential can be related to the electrochemical potential of electrons on an absolute scale, also referenced to electrons in vacuum, as discussed in the electrochemical literature, up to an additive constant.[51] For a meaningful description of the thermodynamics of the electrochemical reactions, the drain potential must be defined versus a reference electrode, because this potential governs the driving force of the redox processes at the electrode–electrolyte interface. Consequently, E_d and V_d differ only by the choice of reference system.

In our system, redox processes occurring in PEDOT:PSS led to a change in the PEDOT:PSS potential measured against the saturated calomel electrode, following the standard electrochemical measurement setup. This variation is induced by the electrochemical reactions taking place at the gate electrode upon applying V_g. Consistently, we observe a substantial change in E_d even when the imposed I_d is equal to zero (see Figure 3C). It is worth noting that the small current flowing in the ionic circuit induces only a minor adjustment of V_d in order to enforce $I_d = 0$.

Because these redox reactions affect the potential of the entire channel when measured versus the reference electrode, they induce changes in both E_d and Es. By definition, since V_d is measured with respect to the source, one can write $V_d = E_d - E_s$. As the electrochemical processes involving PEDOT influence E_d and Es in a similar way, this contribution largely cancels out in the measured V_d. This is

due to the low V_d value (triggered by low applied drain current), that enables symmetrical/uniform channel doping/dedoping.[57] As a result, the variations of V_d are mainly governed by the current flowing through the channel and by the changes in channel resistance associated with the modulation of charge carrier density. By tuning I_d, it is therefore possible to partially control the evolution of V_d.

Figure 3A shows the slope of V_d versus I_d when the applied I_d is −10 μA. Because this current is very small, the resulting change in V_d remains limited and significantly smaller than the corresponding variation of E_d, even when compared with the other experiments reported in this work. For this reason, we observe a magnification of the E_d slope when compared to V_d slope in this condition. We have revised the text accordingly to clarify the relationship between V_d and E_d and to address the reviewer's comment.

SI 2 - Preparation and characterization of PANI-based ITO actuator

The goal of introducing a second electrochromic material in the color-changing measurements was to confirm the versatility and functionality of the procedure. Since the aim of the second attempt was to exhibit how imposing a different I_d can tune the E_d range and variation, PANI was selected as a confirmation material for measurements with an imposed positive I_d.

PANI was electrodeposited on an ITO electrode surface through cyclic voltammetry (CV). The provided set-up included a three-electrode structure, with the ITO electrode being the WE, while SCE represented RE and a Pt wire the CE, with a solution of H_2SO_4 0.1 M and aniline 0.05 M. The electrodeposition was conducted in a potential window from -0.2 to +1.0 V at 50 mV/s for 15 cycles, as shown in Fig. SI 17a. The final product was a deep green deposition on the surface of ITO.[58]

Consequentially, the spectroelectrochemical characterizations were conducted in a cuvette with a similar three-electrode structure, in H_2SO_4 0.1 M. The amperometries were operated from -0.2 to +0.8 V and the absorbance spectrum was acquired after 60 s. The characterization highlighted the electrochromic behavior of the material, indicating a blue-violet coloration at +0.8 V, due to pernigraniline state, and a deep green color from +0.6 to +0.2 V, typical of its emeraldine state. The green color fades at potentials lower than +0.2, since PANI reduces towards its leucoemeraldine state, as shown in Fig. SI 17b.[59]

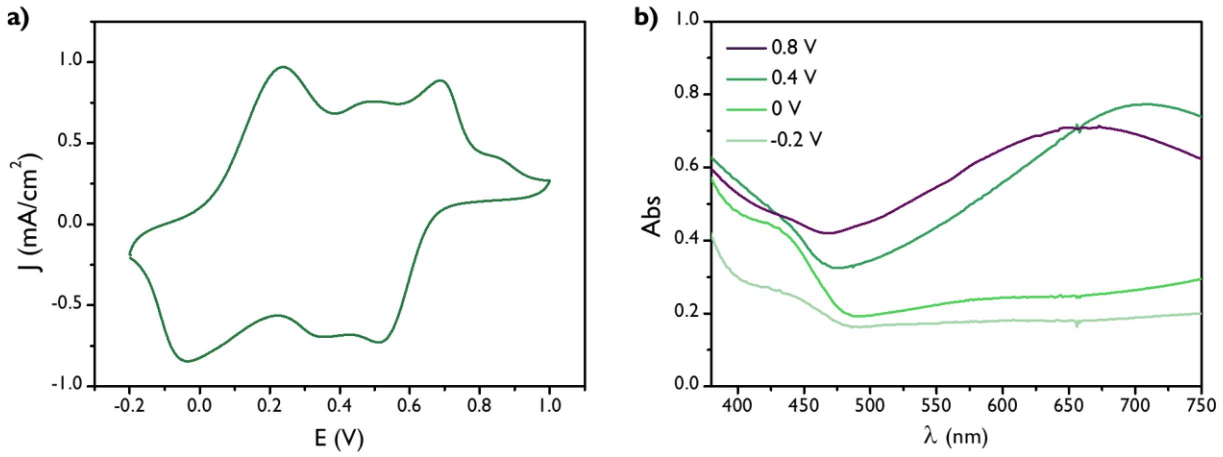

Fig. SI 19 a) Electrochemical deposition of PANI through cyclic voltammetry from -0.2 to +1.0 V vs. SCE, with the resulting currents expressed over ITO glass area; b) absorption spectrum of PANI deposited on ITO glass at +0.8 V (purple), +0.4 V (green), 0 V (pale green) and -0.2 V (grey).

SI 3 – List of Multimedia content

Multimedia file 1) Video of PB-ITO electrode changing color with dopamine addition, without any further system.

Multimedia file 2) Video of PB-ITO electrode inside a septum with dopamine addition, without any further system.

Multimedia file 3) Video of the dopamine-driven electrochromic actuation, shown in Fig 5a) in pictures and 5b) as V_d variations.

Multimedia file 4) Video of the dopamine-driven electrochromic actuation with an I_d equal to -25 µA in PBS 0.1 M pH 7 with dopamine additions.

Multimedia file 5) Video of the dopamine-driven electrochromic actuation with an I_d equal to -100 µA in PBS 0.1 M pH 7 with dopamine additions.

Multimedia file 6) Video of the dopamine-driven electrochromic actuation with an I_d equal to -50 µA and no GCE as functioning gate electrode in PBS 0.1 M pH 7 with dopamine additions.

Multimedia file 7) Video of the dopamine-driven electrochromic actuation with an I_d equal to -25 µA in PBS 0.1 M pH 7 with dopamine additions with a different operator.

Multimedia file 8) Video of the dopamine-driven electrochromic PANI-based actuation with an I_d equal to -50 µA and in PBS 0.1 M pH 7 with dopamine additions.

References

[57] R. Colucci, H. F. D. P. Barbosa, F. Günther, P. Cavassin, G. C. Faria, *Flexible and Printed Electronics* **2020**, *5*, DOI 10.1088/2058-8585/ab601b.

[58] A. Aynaou, B. Youbi, M. Ait Himi, Y. Lghazi, J. Bahar, C. El Haimer, A. Ouedrhiri, I. Bimaghra, Electropolymerization investigation of polyaniline films on ITO substrate, *Mater. Today Proc.*, Elsevier Ltd, **2022**, pp. 335–340.

[59] Z. A. Boeva, V. G. Sergeyev, Polyaniline: Synthesis, properties, and application, *Polymer Science - Series C* **2014**, *56*, 144.
